# Engineered Cellular Therapies for the Treatment of Thoracic Cancers

**DOI:** 10.3390/cancers17010035

**Published:** 2024-12-26

**Authors:** Spencer M. Erickson, Benjamin M. Manning, Akhilesh Kumar, Manish R. Patel

**Affiliations:** 1 Internal Medicine Residency Program, Department of Medicine, University of Minnesota Medical School, Minneapolis, MN 55455, USA; 2 Division of Hematology, Oncology, and Transplantation, Department of Medicine, University of Minnesota Medical School, Minneapolis, MN 55455, USAkumar786@umn.edu (A.K.)

**Keywords:** CAR-T cells, NK cells, lung cancer, mesothelioma, cell therapy

## Abstract

Thoracic malignancies (lung cancers and malignant pleural mesothelioma) are prevalent worldwide and are associated with high morbidity and mortality. Effective treatments are needed for patients with advanced disease. Cell therapies are a promising approach to the treatment of advanced cancers that make use of immune effector cells that have the ability to mediate antitumor immune responses. In this review, we discuss the prospect of chimeric antigen receptor-T (CAR-T) cells, natural killer (NK) cells, T cell receptor-engineered (TCR-T) cells, and tumor-infiltrating lymphocytes (TILs) as treatments for thoracic malignancies. CAR-T cells and TILs have proven successful in several hematologic cancers and advanced melanoma, respectively, but outside of melanoma, results have thus far been unsuccessful in most other solid tumors. NK cells and TCR-T cells are additional cell therapy platforms with their own unique advantages and challenges. Obstacles that must be overcome to develop effective cell therapy for these malignancies include selecting an appropriate target antigen, combating immunosuppressive cells and signaling molecules present in the tumor microenvironment, persistence, and delivering a sufficient quantity of antitumor immune cells to the tumor. Induced pluripotent stem cells (iPSCs) offer great promise as a source for both NK and T cell-based therapies due to their unlimited expansion potential. Here, we review clinical trial data, as well as recent basic scientific advances that offer insight into how we may overcome these obstacles, and provide an overview of ongoing trials testing novel strategies to overcome these obstacles.

## Simple Summary

Cell therapies have led to remarkable responses in patients with advanced hematologic cancers, but these results have not been as readily reproduced in solid tumor malignancies. Chimeric antigen receptor-T (CAR-T) cells, natural killer (NK) cells, T cell receptor-engineered (TCR-T) cells, and tumor-infiltrating lymphocytes (TILs) are adoptive cell therapies (ACTs) that are under investigation for treatment of multiple solid tumors, including the thoracic malignancies that will be the focus of this review. These therapies have enormous potential to help patients even with advanced and refractory disease, but much work remains to design safe and effective cell therapies for these diseases.

## 1. Introduction

Treatment of thoracic malignancies, such as lung cancer and malignant pleural mesothelioma (MPM), has seen considerable advancements in recent years due to innovations including immune checkpoint inhibitors (ICIs) and targeted therapies. However, outcomes for patients with advanced stage disease remain poor, with lung cancer being the leading cause of cancer-related death globally. Despite these advancements, innovative approaches are needed to improve outcomes for patients afflicted with these malignancies [1,2]. Adoptive cellular therapy (ACT) is an approach that uses donor or autologous effector immune cells (most often cytotoxic T lymphocytes) that are expanded or engineered ex vivo to be more cytotoxic against cancer cells [3,4]. This concept was first explored in clinical trials in the 1980s at the National Cancer Institute [5]. In these early trials, some patients with solid malignancies responded to infusions of autologous lymphokine-activated killer cells [6,7]. Further basic research led to the use of a more potent cell therapy known as tumor-infiltrating lymphocytes (TILs), and impressive responses were seen in patients with metastatic melanoma in an early trial of this new form of immunotherapy [8,9]. Since these initial trials, much progress has been made and several new approaches to ACT are being explored at both the preclinical and clinical stages [10]. One such cell therapy, chimeric antigen receptor T cell (CAR-T) therapy has seen tremendous success in CD19-positive hematologic malignancies, but multiple barriers to an effective CAR-T product for solid tumors remain [11].

This review will focus on using ACT as a treatment for lung cancer and MPM. While clinical trials of cell therapy products in solid tumors have yet to deliver results comparable to those in hematologic cancers [10], recent preclinical advances, the success of TILs in a solid tumor (melanoma) [12], and recent hints of the efficacy of CAR-T cells in early phase trials of solid tumors such as glioblastoma [13,14] and neuroblastoma [15] offer hope that the coming decade will see further advances and eventual clinical translation of cellular immunotherapies in thoracic malignancies and other solid tumors. Advancements in stem cell and genetic engineering techniques in recent years have also allowed for the possibility of more efficient gene editing to create more effective cell therapies, as well as the possibility of “off-the-shelf” products, which have the potential to greatly reduce cost and treatment delays owing to manufacturing times [4]. First, we will discuss target antigens of interest for lung cancer and MPM; then briefly define TILs, CAR-T cells, TCR-T cells, and NK cells; and discuss issues pertaining to the source of cells used for these therapies. We will then highlight the results of the key published clinical trials for each of these cell therapies. Finally, we will explore some of the barriers to effective therapy and the preclinical work that has been carried out to address some of these barriers.

## 2. Target Antigen Selection

Selection of an appropriate antigen target for cell therapies is crucial, not only for efficacy, but also due to the possibility of toxicity related to antigen expression on healthy tissues (on-target, off-tumor toxicity) [5,16,17,18]. Targets for cell therapies fall into three general categories: tumor-associated antigens (antigens expressed at higher levels in cancer cells but that may also be expressed in some healthy tissue, or in which expression is typically restricted to fetal development), tumor-specific antigens (neoantigens that arise due to mutations and are expressed only on cancer cells, as well as viral oncoproteins), and unconventional antigens (antigens derived from non-coding DNA regions or errors in transcription, translation, or post-translational modifications) [3]. For optimal efficacy and safety, the ideal target antigen is both highly expressed and highly specific to cancer cells [3]. Predictive algorithms, immunopeptidomics, and various other methods have been used to identify target antigens [3]. Some of the possible antigen targets for cell therapies in thoracic malignancies are summarized in Table 1 [19,20,21,22,23,24,25,26,27,28,29,30,31,32,33,34,35,36,37,38,39,40,41,42,43,44,45,46,47,48,49,50,51,52,53,54,55,56,57,58,59,60,61,62,63,64,65].

## 3. TIL Therapy

TILs are intratumoral lymphocytes (mostly CD4+ and CD8+ T cells) that are harvested from resected tumors and expanded and activated with cytokines ex vivo prior to infusion into patients [5]. In contrast to the other T cell-based therapies discussed in this review, TILs are a polyclonal population of CD4+ and CD8+ T cells that are selected on the basis of their presence in the tumor microenvironment rather than their ability to recognize a specific target antigen [5,8,10]. Importantly, a higher proportion of TILs has been associated with better survival in patients with NSCLC and MPM [66,67,68]. Unlike the other cell therapies discussed in this review, TILs have proven efficacious in a phase 3 trial of a solid tumor (melanoma) [12].

## 4. CAR-T Cell Therapy

CAR-T cells are T lymphocytes which have been genetically engineered to express a chimeric antigen receptor (CAR) which includes an extracellular single-chain variable fragment (scFv) that binds a surface antigen of interest in a non-MHC-restricted fashion, a transmembrane domain, and a signaling domain [5,16]. Multiple generations of CAR-T cells have been developed to address toxicity concerns and to optimize T cell signaling and cytotoxicity [16]. Intricate details of CAR-T cell design and engineering are beyond the scope of this article and are reviewed elsewhere [16].

## 5. TCR-T Cell Therapy

TCR-T cell therapy is a form of ACT that utilizes genetic transfer of antigen-specific TCRs into autologous T cells for the treatment of cancer [17,69]. T cells reactive against antigens/epitopes of interest are isolated from patient samples, the identified TCR is sequenced, and is subsequently introduced into patient T cells through various genetic engineering techniques, such as CRISPR-Cas9 or viral vectors [17]. The TCR-T cells can then be infused into HLA-compatible patients [17]. The concept is otherwise very similar to CAR-T cells, with the notable exception that TCR-T cells can target both intracellular and extracellular antigens presented in the context of self-MHC and thus require functional antigen presentation machinery and are subject to HLA-restriction, as opposed to CAR-T cells which target surface antigens via their CARs [5,17]. In addition, TCR-T cells signal through the traditional TCR signaling pathway that more closely matches normal physiology, which offers potential advantages over the artificial signaling complex utilized by CAR-T cells [16,17]. As they are also T cells, TCR-T cells can easily be combined with many of the strategies described later in this review that are meant to augment CAR-T cell activity. An additional advantage of TCR-T cells over CAR-T cells is increased sensitivity, as TCR-T cells mediate intracellular activation with orders of magnitude higher sensitivity than CAR-T, which requires additional engineering to approximate the T cell activation on binding to its receptor [70].

## 6. NK Cell Therapy

NK cells are lymphocytes of the innate immune system that can kill tumor cells via the formation of an immune synapse with the target cell and subsequent release of cytolytic granules containing perforin and granzyme, promotion of several ligand-mediated cell death programs, and through antibody-dependent cellular cytotoxicity (ADCC) via engagement of the Fc portion of an antibody with their CD16 receptors [71]. They also promote immune responses against tumors by secreting interferon-γ (IFN-γ) and other cytokines and chemokines [71]. NK cells have several activating receptors (NKG2C, NKG2D, activating killer immunoglobulin-like receptors (KIRs), etc.) which recognize stress-induced ligands or viral ligands, as well as inhibitory receptors (inhibitory KIRs, NKG2A, siglecs, etc.) that suppress NK cell activity and prevent autoimmunity [71,72]. NK cells also have the unique capacity for “missing-self recognition”, whereby NK cells detect and kill cells with deficient self MHCI expression, a tactic many cancers employ to evade detection by cytotoxic T cells [72]. In contrast to T cells, NK cells undergo a dynamic education/licensing process, whereby NK cell activity increases when more inhibitory receptors recognize self MHCI and decreases when activating receptors recognize self-antigens [71,72]. NK cells also offer potential safety advantages over T cell-based therapies, namely a lower incidence of CRS and immune-effector cell-associated neurotoxicity syndrome (iCANS) and a much lower risk of graft-versus-host disease (GVHD) [18]. Given these theoretical advantages over T cell-based therapies, NK cells have gained significant attention in recent years as an alternate cell therapy platform and have been explored in early phase clinical trials in a wide range of malignancies, with early evidence of some efficacy in hematologic cancers [36,73]. However, clinical efficacy of NK cell therapies has been limited to date, especially in solid tumors [18,36].

## 7. Cell Sources for ACT

The predominant source of cells used in ACT to date has been an autologous source. The main advantage of using an autologous source is there is little concern for rejection or GVHD if using autologous cells. In hematologic malignancies, all FDA-approved cell therapy utilizes an autologous source. The main drawback of autologous cells is the extensive time and cost in manufacturing an individual cell therapy product for each patient. This often results in a lag of 4–6 weeks from the time of leukapheresis to obtain the cells and the eventual infusion of the therapeutic product. Most patients cannot wait this long to receive treatment, and thus, this novel form of treatment relies on having some effective therapy to stabilize disease while awaiting manufacture.

Induced pluripotent stem cells (iPSCs) have transformed cellular immunotherapy by enabling the reprogramming of somatic cells into a pluripotent state [74,75,76,77]. This breakthrough provides a renewable and expandable source of immune and blood cells, including T cells, NK cells, and macrophages [78,79,80,81]. iPSCs can be derived from cancer patients, genetically modified to target diseased cells, and used for transplantation without the risk of GVHD. The use of iPSC banks or hypoimmunogenic iPSCs offers cost-effective, off-the-shelf therapies for a wide patient population, streamlining treatment logistics. These advancements hold promise for revolutionizing the treatment of hematological and solid tumors like thoracic cancers.

Traditional autologous CAR-T cell therapies have advanced immunotherapy but are hindered by challenges like suboptimal T cell fitness, limited patient material, and costly manufacturing. iPSC technology addresses these issues by deriving therapeutic T cells from healthy donors. This process includes reprogramming somatic cells, differentiating them into hematopoietic progenitor cells (HPCs), and culturing under conditions that mimic the thymic environment. The resulting iPSC-derived T cells undergo CAR or TCR engineering for targeted tumor antigen recognition, with successful reprogramming and re-differentiation achieved for multiple antigens, including MART-1 [82], Nef [83], MR-1 [84], LMP1/2 [85], and WT-1 [86]. These iPSC-derived T cell products offer scalable, consistent production, enhanced cellular fitness, and multi-antigen targeting, reducing the risk of GVHD [85,87,88]. To reduce allo-reactivity, iPSCs with known TCR specificity against common antigens are used to generate T cells, though HLA-matching remains necessary; iPSC-derived T cells have shown tumor control in preclinical models, and a first-in-human trial with iPSC-derived TCR-T cells is underway. NCT06241456 is investigating iPSC-derived CAR-T cells directed against HER2 (arm A). Interestingly, this study will explore the combination of anti-HER2 CAR-T cells with cetuximab, taking advantage of the engineering of a non-cleavable CD16 into the T cell product to mediate antibody-dependent cytotoxicity (arm B) in patients with EGFR-expressing NSCLC, head and neck cancer, and colorectal cancer.

iPSC-derived CAR-T cells may provide an ideal platform to allow for the ability to develop a CAR-T product with multiple genetic alterations designed to optimize CAR-T cell efficacy [11]. This platform makes “off the shelf” cell therapy products feasible, and such a product would help to avoid delays in treatment related to manufacturing time [4]. Advances in genetic engineering techniques, such as the recently characterized CRISPR-Cas13d system, that allow for precise regulation of expression of multiple genes simultaneously will hopefully make this feasible in future generations of CAR-T cell products [89]. Another promising approach that is under investigation is using allogeneic CAR-T cells derived from donors with genetic disruption of endogenous TCRs that would minimize the risk of GVHD owing to the lack of endogenous TCR (though this risk is not completely eliminated, as mismatching with other HLA alleles could still result in GVHD). Such an approach would also allow for the development of a universal “off the shelf” product [90]. Multiple clinical trials are currently investigating allogeneic CAR-T cells: NCT05239143 is investigating allogeneic CAR-T cells targeted against MUC1-C in patients with advanced MUC1-C expressing NSCLC and other advanced solid tumors; and NCT05795595 is investigating allogeneic CAR-T cells targeted against CD70 in patients with advanced MPM and other advanced solid tumors.

NK cells used in cell therapy products can be derived from patients or donors from one of several sources (reviewed in detail by Berrien-Elliot et al., 2023), including peripheral blood (PB), hematopoietic stem cells (HSCs), umbilical cord blood (CB), and iPSCs [18]. PB NK cells allow for an off-the-shelf approach, less manufacturing time, and have good effector function, but are limited by variability in donor NK cells and limited potential for ex vivo expansion [18,91]. NK cells derived from HSCs have good ex vivo expansion potential, and donor variability is less of a concern, but they are limited by low effector function [18,91]. CB NK cells have moderate expansion potential and can be easily accessed via global cord blood banks but are limited by the need for a lengthy differentiation process and variability among donors [18,91]. The immortalized NK lymphoma cell line NK-92 is another source of NK cells for immunotherapy that has advantages including lack of inhibitory KIR expression and ability to be easily expanded and engineered ex vivo, but are limited by the need for irradiation prior to infusion and cannot mediate ADCC because of a lack of CD16 expression [91].

Another important consideration is the distinction between autologous and donor derived/allogeneic NK cells. This is due to the observation that a mismatch between inhibitory KIRs on donor NK cells and recipient tumor cells may lead to improved recognition of tumor cells and induce a “missing self” response [18,92]. This finding of improved outcomes with a mismatch of donor KIR and recipient ligands has been termed the “graft-versus-tumor effect” and has been associated with improved responses in patients with hematologic malignancies who have received hematopoietic stem cell transplants [18,92].

Each source of NK cells has potential advantages and drawbacks; however, similar to CAR-T cells, NK cells derived from iPSCs seem to hold the greatest potential moving forward owing to their potential for efficient large-scale production of multigene edited NK cells [18]. Human embryonic stem cell (hESC)/iPSC-derived NK cells, like CB- and PB-NK cells, eliminate target cells by releasing perforins and granzymes, secreting proinflammatory cytokines such as IFN-γ and tumor necrosis factor α (TNF-α), and triggering apoptosis via TRAIL and Fas-FasL interactions [93,94]. iPSC-derived NK cell therapy offers a promising therapeutic avenue by combining the innate cytotoxicity of NK cells with the enhanced expansion potential of iPSCs. These cells, derived from healthy donors, are produced through directed differentiation using cytokines and growth factors, resulting in functional cytotoxic NK cells [95,96]. Recent advancements in feeder-free protocols have improved the scalability of this process [97,98,99,100]. iPSC-NK cells can be engineered with CARs for HLA-independent tumor targeting and offer a safer profile with lower risks of GVHD, CRS, and neurotoxicity than T cells. In ovarian cancer xenograft models, iPSC-NK cells showed similar cytotoxicity to PB-NK cells, while another study demonstrated that iPSC-NK cells had superior cytotoxicity against ovarian, colon, and breast cancer cell lines [99,101]. Ongoing research aims to enhance their efficacy, persistence, and tumor infiltration, making iPSC-derived NK cells a potential next-generation immunotherapy, especially for solid tumors. A significant benefit of iPSCs is genetically engineering NK and T cell effector responses for immunotherapy. *CISH* deletion in iPSC-derived NK cells is a recent advance. The *CISH* gene produces cytokine-inducible SH2-containing protein (CIS), which inhibits cytokine signaling and signal transduction. Studies in *CISH*^−/−^ mice show that NK cells without *CISH* have better tumor control and IL-15 sensitivity, especially in lung metastasis models [102,103]. This suggests that *CISH* deletion could enhance NK cell-based cancer therapies.

## 8. TIL Trials

Few trials have reported results of TIL therapy in patients with lung cancer, and none have reported results in patients with MPM. Results to date have been modest, with a few notable exceptions of patients achieving impressive responses [104,105,106,107,108,109,110,111]. The first trial to report results of TIL therapy in patients with thoracic malignancies was conducted by Kradin and colleagues (1987) [104]. They observed a reduction in tumor size in five of seven patients with metastatic lung adenocarcinoma; however, none of the patients achieved a partial response (PR). While ^111^indium-oxide radiolabeled TILs were not detected above background with γ-camera imaging in sites of primary tumor or metastases, biopsied tumor samples and control tissue revealed increased radioactivity in tumor compared to control tissue, suggesting TILs preferentially migrated to tumor tissue. Notably, patients in this trial did not receive exogenous cytokine support after infusion of TILs. A follow-up trial of TILs plus continuous infusions of IL-2 in patients with advanced NSCLC, melanoma, or renal cell carcinoma was published in 1989 [105]. Results were disappointing in NSCLC patients, with zero complete responses (CRs) or PRs seen (three out of eight patients with no response, and five out of eight patients with PD). However, 3/13 patients with melanoma and two of seven patients with renal cell carcinoma (RCC) did achieve a PR. It is unclear whether the lack of response in the NSCLC patients was due to lower quality TILs or intrinsic differences in tumor biology between NSCLC and RCC and melanoma. Interestingly, cell dose and IL-2 dose did not correlate with clinical responses in this trial. As in their prior trial, radiolabeled TILs were not detected within tumors with γ imaging.

Two pilot studies by the same group in Italy in the mid-1990s showed that TIL therapy was safe, feasible, and possibly effective in patients with stage III resected NSCLC [106,107]. These encouraging results prompted them to conduct a larger randomized trial, which was published in 1996 and remains the largest trial of TIL therapy in patients with NSCLC conducted to date [108]. This trial tested autologous TILs plus IL-2 in patients with stage 2, 3a, or 3b NSCLC deemed to have potentially resectable disease and reported encouraging results among the subset of patients with stage 3b disease. Patients were stratified by disease stage and randomized to active treatment with TILs and IL-2 or control. In total, 131 patients were enrolled and had their tumors resected, 18 of which had no TILs grown on cultures obtained from their tumors. The remaining 113 patients were randomized to TIL infusion or control. Patients with stage 2 disease were randomized to TIL infusion or no additional therapy. Patients with stage 3a or 3b disease were randomized to TIL infusion plus radiotherapy (RT) or RT plus chemotherapy. Among all patients receiving TIL infusion, median overall survival (OS) was 22.4 months, which was significantly greater than the median OS of 14.1 months for control (*p* < 0.05). For patients with stage 3b disease, median OS was 23.9 months in the TIL plus RT group as compared to 7.3 months for the control group (*p* < 0.01). For patients with either stage 3a or 3b disease, median OS was 22.4 months in the TIL plus RT group vs. 8.9 months for the control group (*p* < 0.01). No statistical benefit was observed for patients with stage 3a disease or patients with stage 2 disease compared to their respective controls. The treatment was well-tolerated, with only minor symptoms such as fever, chills, and nausea reported. Given only the patients with 3b disease seemed to benefit, this suggests that more work remains to carefully identify which patients are most likely to benefit from TILs. A follow-up phase 2 study by the same group among patients with incompletely resected stage 3 NSCLC yielded disappointing results [109]. In total, 13 patients were treated with TILs plus IL-2, followed later by cisplatin and etoposide, as well as RT. Unfortunately, there was no statistical difference in either median PFS or median OS between these patients and a comparison group (n = 20) that received chemotherapy and RT, but no TILs. Importantly, TILs were given prior to chemotherapy, and the authors note that this sequencing may be to blame for the seeming lack of benefit observed.

Creelan and colleagues (2021) conducted a phase 1 trial of autologous TILs plus IL-2 in patients with metastatic NSCLC patients who had previously progressed on nivolumab [110]. Patients were given nivolumab infusions until PD was noted, at which time they received cyclophosphamide and fludarabine for lymphodepletion. They were then given TILs and IL-2 and were continued on nivolumab every 4 weeks. In total, 20 patients were enrolled and 16 patients received TIL infusion; 11/16 patients had radiographic evidence of tumor regression 1 month after TIL infusion; 13 patients were considered evaluable for response, 6 of whom had a radiographic response, including patients with an unconfirmed response; and 3/13 patients had a confirmed response. Remarkably, two of these patients had a continued CR at 1.5 years. Of note, one patient with an EGFR mutation had a CR and in vitro assays performed after TIL treatment in which T cells were stimulated with peptides, which revealed that this patient had 19 antigen-specific T cell clonotypes. Another interesting finding in this study was that one patient who achieved a CR had a low tumor mutational burden. Finally, both T cell immunoglobulin and mucin-domain-containing-3 (TIM-3) and T cell immunoreceptor with Ig and ITIM domain (TIGIT) expression was high in all TILs in this trial, suggesting a potential strategy of blocking these targets in future trials. The most recently published trial was conducted by Schoenfeld and colleagues (2024) [111]. This was a phase 2 trial of autologous TILs in patients with metastatic NSCLC who had experienced progression after prior anti-PD-1/PD-L1 ICI. Results were encouraging in this trial, with 19/28 patients who received TIL infusion experiencing a reduction in tumor burden and an ORR of 21.4% (95% CI, 8.3–41.0). Interestingly, patients achieving a response included those with driver mutations. Another notable finding was one patient who achieved a PR had both an STK11 and KEAP1 mutation.

While larger clinical trials will be informative, several key points can be taken from the trials of TILs conducted to date. To start, it is now well known that adequate lymphodepletion prior to TIL infusion and use of cytokines such as IL-2 to expand TILs after infusion are beneficial and should be standard components of any future trial [5]. It is likely that better responses would have been seen in the earlier trials had these strategies both been employed. However, direct comparison of response rates and clinical outcomes with the more recent trials is challenging, as these trials included more heavily pretreated patients with more advanced disease. Another consideration is the above trials inconsistently characterized several important phenotypic characteristics of infused TILs that may be key to determining why some patients achieved clinical responses and others did not. Preclinical studies have effectively utilized single-cell RNA sequencing and machine learning approaches to identify TILs with phenotypic properties more likely to lead to clinical responses and may underlie the reasons why select patients have achieved remarkable regressions of advanced tumors, while others do not respond [112,113,114]. Thus, future trials should include in their protocols plans to comprehensively characterize the epitope specificity, transcriptional profile, and other phenotypic properties of the TILs used. Such efforts are already underway, as Chiffelle et al. (2024) used single-cell RNA sequencing and TCR sequencing to follow T cell clonotypes over time in patients with metastatic melanoma who received TIL therapy [115]. They found several key differences between patients who responded to TIL therapy and those who did not. Notably, patients who achieved clinical responses had tumors with a greater proportion of tumor-reactive TILs prior to treatment. After TIL infusion, their tumors were infiltrated with T cell clonotypes arising from the tumor, while these clonotypes mostly remained in the bloodstream in patients who did not respond. These results suggest that patients with tumors that have a greater proportion of tumor-reactive TILs in biopsy specimens or resected tumor samples are ideal candidates for TIL therapy. Another consideration for future trials is to consider recruiting patients with higher neoantigen loads, as this has been associated with response to TIL therapy in patients with melanoma [116]. These studies will help to refine inclusion criteria in future clinical trials.

## 9. CAR-T Cell Trials

Though there have been many studies of CAR-T therapy in the preclinical space, very few clinical trials have completed or reported data in thoracic oncology [39,117,118,119,120,121,122]. The bulk of clinical data in thoracic oncology as it relates to CAR-T cells are in the mesothelin (MSLN)-targeted CAR-T cells. MSLN is commonly expressed in the majority of epithelioid mesothelioma and has been a therapeutic target of interest in this disease for >20 years. MSLN-targeted CAR-T cells were one of the earliest CAR-T cell therapies used in solid tumors with the first clinical trial initiated in 2011. Of note, MSLN is also highly expressed in pancreatic cancer, ovarian cancer, and NSCLC, and thus, most of the clinical trials have included other tumor types as well as MPM. The first trial was a CAR that was designed using the murine-derived SS1 antibody against MSLN [123]. The T cells were electroporated with MSLN RNA to express the CAR in T cells transiently. Multiple infusions of MSLN CAR-T cells were allowed. While there were no objective responses (ORs), the toxicity was minor, with the exception of one severe infusion reaction that was attributed to the murine scFv antibody. Encouragingly, the investigators were able to identify CAR-T cells in the tumor after therapy. The next trials used a lentiviral transfection of the SS1 CAR to allow for stable anti-MSLN CAR expression on the T cells [124]. These cells were again well tolerated, but minimal antitumor efficacy was noted. Here, cyclophosphamide lymphodepletion was employed. Though there was evidence of enhanced expansion of CAR-T cells after lymphodepletion, CAR-T persistence was not improved by using lymphodepleting chemotherapy. Notably, antibodies against the CAR were detectable in 8/14 patients. A fully humanized anti-MSLN CAR-T was developed to overcome this limitation. A phase 1 trial has recently completed accrual at the University of Pennsylvania, recruiting patients with NSCLC, MPM, and ovarian cancer; however, the results of this trial have not yet been published.

Adusumilli and colleagues (2021) used an MSLN CAR-T cell incorporating a suicide-inducible Caspase 9 switch in case of severe toxicity. In this trial, MSLN CAR-T was delivered via a pleural catheter into the pleural space of patients with pleural malignancies (MPM, metastatic lung cancer, and metastatic breast cancer) [119]. In preclinical studies prior to this trial, it was noted that intrapleural administration was associated with better antitumor activity than intravenous administration. For patients with MPM, there was also a cohort of 18 patients treated with pembrolizumab 4 weeks after CAR-T injection to mitigate PD-1 expression and subsequent loss of function of CAR-T that was observed in preclinical models [125]. Intrapleural delivery of MSLN CAR-T was safe with no dose-limiting toxicities observed. CRS occurred in 26% of patients, though all were grade 2 or less. There was no on-target/off-tumor toxicity. Most of the grade 3 or higher toxicity was attributed to lymphodepleting chemotherapy. In total, 16 of these patients had measurable disease and were assessed for best overall response, with 2/16 patients achieving a PR, 6 patients demonstrating some degree of tumor regression without achieving a PR (most > 6 months), and 5/16 patients experiencing PD. Median OS from the time of CAR-T cell infusion among these patients was 23.9 months (95% CI: 14.7 months—unable to estimate) and 1-year OS was 83% (95% CI: 68–100%).

More recently, gavo-cel, an MSLN-targeted T cell receptor fusion construct (TRuC) T cell was developed and was investigated in a phase 1/2 trial by Hassan and colleagues (2023) [126]. The TRuC used in gavo-cel is a humanized scFV against MSLN integrated into the TCR, leaving the downstream signaling function of the TCR intact. Gavo-cel was clinically active and ORs were seen in 20% (6/30) of patients, including one MPM patient with a durable response lasting approximately 1 year. Virtually all patients enrolled had some degree of tumor regression. Patients who received lymphodepletion had deeper responses, but unfortunately, most of the responses were short-lived. Notably, though PD-1 expression on gavo-cel was minimal at onset of treatment, PD-1 expression increased in the nine patients that had longitudinal data available, suggesting that PD-1 expression may have led to blunted antitumor activity over time. CRS was common in this study occurring in 78% of the patients (grade 3/4 in 19% and 6%). There were a few cases of on-target/off-tumor toxicity with two cases of pleuritis, two cases of pericarditis, and one case of peritonitis (all grade 3).

Based on the above studies, MSLN-targeted CAR-T cells remain a promising approach for treatment of MPM and, in particular, intrapleural delivery is appealing for MPM where bloodborne metastasis occurs relatively late in the disease process. Outside of MSLN, few trials have reported data. Two studies from China have evaluated EGFR-targeted autologous CAR-T cells in NSCLC [117,120]. The main difference between the two studies is that one included lymphoconditioning with cyclophosphamide, while the other did not. Though rare responses were seen, they were not durable. Notably, the toxicity was fairly minor. In spite of choosing a target that has expression in many normal tissues, the on-target/off-tumor toxicity was quite minor. Use of conditioning chemotherapy was associated with increased persistence of CAR-T cells.

Receptor tyrosine kinase-like orphan receptor (ROR1) has been seen as a promising target of CAR-T therapy for NSCLC. ROR1 is commonly expressed during embryonic development, but its expression is virtually tumor-specific in adults. A phase 1 trial by Specht et al. (2018) tested autologous CAR-T cells directed against ROR1 for patients with ROR1-positive triple-negative breast cancer or NSCLC and observed a mixed response among the two NSCLC patients [122]. The treatment was safe with minimal CRS observed. An additional trial using Lyl797, a ROR1-targeted CAR-T cell, enrolled patients with triple-negative breast cancer or NSCLC (NCT05274451). This CAR-T cell was designed to overexpress c-Jun to overcome T cell exhaustion. To date, 20 patients have been enrolled; however, only 4 were NSCLC patients. Still, the results were recently disclosed via press release that there was a 40% ORR among the patients who received the 150 × 10^6^ cells dose level (n = 5). There was increased toxicity with Lyl797; in particular, CRS was seen in 60% of patients (all grade 1 or 2). Perhaps more concerning, pneumonitis was seen in 22% of patients with lung involvement with cancer. None of the cases were seen in patients without lung involvement. The study is ongoing enrolling patients with NSCLC, and the study is now instituting dexamethasone pre-treatment in an effort to mitigate pneumonitis and CRS [127]. The above results with Lyl797 are preliminary, and it remains to be seen if this is going to be a successful strategy for patients with NSCLC given that most of these patients will have disease in the lungs and many come to the trial with prior history of underlying lung disease.

Delta-like ligand 3 (DLL3) is a well-validated therapeutic target in small cell lung cancer (SCLC). Recently, T cell engager molecule targeting DLL3, tarlatamab, has been FDA approved for SCLC, and antibody-drug conjugates (ADCs) have been shown to have antitumor activity against SCLC in early trials [128]. Byers et al. (2022) reported results of a phase 1 trial of CAR-T cells directed against DLL3 in patients with relapsed or refractory small cell lung cancer and found a possible signal of efficacy and no concerning safety signals [39]. Five patients received CAR-T cells. The most serious AE was grade 3 pneumonitis in one patient. Four patients were evaluated for response, with one patient experiencing a PR, two patients experiencing SD, and one patient experiencing PD. Median PFS was 3.7 months (range of 1.1–6.7 months) and median OS was 7.4 months (range of 4.6–18.9 months).

The coming years will hopefully yield much more data as several studies are currently underway to build upon the foundation. At this time, it is too early to make any assertions as to the best antigen targets for CAR-T therapy. Though speculative, it appears that the ancillary edits to T cells that will enhance tumor penetration and T cell persistence may be ultimately the most important factor in determining the activity of these CAR-Ts. As has been seen with the anti-MSLN CAR-T therapy, several iterations of CAR-T trials may be necessary to obtain an active product and continuous refinement of the best approaches.

## 10. TCR-T Cell Therapy Trials

Very few trials of TCR-T cells that have included patients with thoracic cancers have reported results to date [129,130,131]. The first trial was a phase 1 dose-escalation trial of affinity-enhanced MAGE-A10 TCR-T cells in patients with MAGE-A10-expressing advanced NSCLC [129]. In total, 7 of 11 patients in the trial were evaluable for response. Five out of seven patients had SD, and two out of seven patients had PD. One patient received a second infusion of TCR-T cells and then achieved a PR. Median PFS was 58 days (range of 1 to 89 days). Median OS was 132 days (range of 10 to 458 days). There were two grade 5 AEs deemed unrelated to the study treatment. Notably, the TCR-T cells persisted in the peripheral blood of patients for up to 12 months, and tumor infiltration of these cells was detected in two of four biopsy samples. While no severe treatment-related AEs were noted in this trial, great efforts should be made to ensure the safety of affinity-enhanced TCR-T cells in any future trial, as this strategy has an inherently increased risk of off-target toxicity owing to the fact that it bypasses the safety mechanism of thymic selection [132]. An unfortunate example of this risk was demonstrated in early trials of affinity-enhanced TCR-T cells directed against HLA-A*01-restricted MAGE-A3 in patients with MAGE-A3 expressing tumors in which two patients died of cardiac toxicity that was eventually found to be related to unanticipated cross-reactivity to a titin peptide [133,134].

A second phase 1 trial by Hong and colleagues (2023) tested affinity-enhanced MAGE-A4 TCR-T cells in patients with relapsed or refractory solid tumors that expressed MAGEA4 [130]. In total, 38 HLA A*02-positive patients received TCR-T cell infusion after lymphodepletion. Two of these patients had NSCLC, one of whom achieved a PR. Morelli and colleagues (2023) conducted a phase 1/2 trial of TCR-T cells directed against driver mutations in KRAS, TP53, and EGFR [131]. Patients with NSCLC and other solid tumors with one of the respective driver mutations were eligible to enroll. As for preliminary reporting, three patients had received TCR-T cells with expected safety profile. One PR was seen in the lone patient with NSCLC treated with an HLA A*11:01-restricted TCR targeting G12D-mutated KRAS peptide. One patient had SD, and one patient had PD. All three patients had CRS (grade 3 or less), and the treatment was otherwise well-tolerated, with no dose-limiting toxicities observed.

## 11. NK Cell Therapy Trials

Compared to the T cell-based therapies described above which have been explored in patients with thoracic cancers for decades, trials of NK cells in these malignancies are a much more recent venture, with the first trial reporting results in 2010 [60,135,136,137,138,139,140,141,142,143]. The first trial to report results was a phase 1 trial conducted by Iliopoulou and colleagues (2010) which tested allogeneic NK cells that had been activated and expanded ex vivo with IL-15 and hydrocortisone [135]. Patients with advanced NSCLC were given 2–4 infusions of NK cells between cycles of chemotherapy. PFS among these patients was 5.5 months (range of 1–22 months). While responses were relatively rare, the lack of serious AEs established the safety of NK cell therapy in patients with NSCLC. A phase 2a trial by Yang et al. (2013) utilized autologous NK cells and concurrent docetaxel in patients with advanced NSCLC [136]. The treatment was well-tolerated, but efficacy was modest with an ORR of 10.5% and a median PFS of 2.9 months (range of 1.3–6.4 months), not more than would be expected with docetaxel alone. Tonn et al. (2013) administered NK cells derived from the cell line NK-92 to patients with advanced cancer [137]. In this trial, two of three patients with SCLC had a mixed response (MR) and OS of these patients ranged from 218 days to 388 days. The only NSCLC patient in the trial achieved SD and had an OS of 707 days. There was some evidence of persistence, as NK-92 cells were detected at 48 h post infusion in two patients. Lin and colleagues (2017) carried out a prospective study of allogeneic NK cells plus CT-guided percutaneous cryoablation of tumors versus CT-guided percutaneous cryoablation alone in patients with advanced NSCLC [138]. The treatment was well-tolerated, and NK cells seemed to be of benefit, with a response rate of 63.3% in the combination group vs. 43.3% in the cryoablation monotherapy group (*p* < 0.01), a disease control rate (DCR) of 83.3% in the combination group vs. 70% in the cryoablation monotherapy group (*p* < 0.05), and a CR in 7/30 patients in the combination group vs. 4/30 patients in the cryoablation monotherapy group (*p* > 0.05). Multhoff et al. (2020) conducted a trial of autologous NK cells stimulated ex vivo with low-dose IL-2 and membrane-bound Hsp70 (mHsp70) peptides in patients with unresectable NSCLC with expression of mHsp70 after radiochemotherapy [60]. There were no AEs felt to be related to the NK cells and, while not statistically significant, some evidence of efficacy was seen, as the 1-year probability of PFS was 67% (95% CI, 19–90%) in the treatment group as compared to 33% (95% CI, 5–68%) in the control group (*p*-value = 0.36).

Several trials have also explored combining NK cell therapy with ICIs. Jia et al. (2022) conducted a pilot study of autologous NK cells in combination with the anti-PD-1 monoclonal antibody sintilimab in patients with advanced NSCLC who had previously received platinum-based chemotherapy [139]. The treatment was well-tolerated and showed some hints of efficacy, with an OS of 17.7 months (range of 2.3–25.9+ months), ORR of 45%, and a median PFS of 11.6 months (1.33–25.9+ months). Though this study did not have a control arm, the ORR and PFS were much higher than would be expected with monotherapy anti-PD1 therapy, and thus, the results suggested potential for efficacy. Another trial (Lin et al., 2020) tested NK cells plus pembrolizumab vs. pembrolizumab monotherapy in patients with advanced NSCLC and found the combination treatment was superior to pembrolizumab monotherapy with respect to median OS, median PFS, and ORR [140]. The treatment was well tolerated, with no grade 4 AEs reported. In the combination group, median OS was 15.5 months, median PFS was 6.5 months, and ORR was 36.4%. Of note, multiple infusions of NK cells was associated with an OS benefit. Kim et al. conducted a phase 1/2a trial of autologous NK cells plus pembrolizumab vs. pembrolizumab monotherapy in patients with advanced NSCLC who had previously failed platinum-based chemotherapy and had a PD-L1 tumor score of at least 1% [141]. The NK cells were well-tolerated, and the group that received NK cells and pembrolizumab had better outcomes compared to pembrolizumab monotherapy, with a median PFS in the NK cells plus pembrolizumab group of 6.2 months (95% CI, 1.4 months—not listed) as compared to 1.6 months (95% CI, 0.6–4.7 months) in the patients who received pembrolizumab monotherapy and a 1-year survival rate of 66.7% in the NK cells plus pembrolizumab group compared to 50% in the pembrolizumab monotherapy group. An extension of this same trial confirmed a long-term benefit in the NK cells plus pembrolizumab group, with a 2-year survival rate of 58.3% as compared to 16.7% in the pembrolizumab monotherapy group and a HR for survival of 0.32 (95% CI, 0.1–1.08, *p* = 0.066) for the NK cells plus pembrolizumab group compared to the pembrolizumab monotherapy group [142]. Interestingly, there was no PFS or OS benefit for the high-dose NK cell group compared to the low-dose NK cell group. A phase 1 trial by Patel and colleagues (2022) took a novel approach by using allogeneic “off-the-shelf” NK cells derived from iPSCs in combination with IL-2 and either atezolizumab, nivolumab, or pembrolizumab after lymphodepletion with cyclophosphamide and fludarabine in patients with advanced solid tumors that were refractory to or relapsed following prior PD-1/PD-L1 ICI therapy [143]. The treatment was well-tolerated overall, and among the six NSCLC patients included in the efficacy analysis, one patient had an ongoing PR at 12 months, three patients had SD, and the median duration of disease control was 8.6 months [143]. The encouraging results from this trial established the safety of iPSC-derived NK cells and will hopefully encourage further clinical trials that utilize the iPSC platform.

Responses were seen with several sources of NK cells, so it is not clear whether one approach may offer superior efficacy. Further research is also needed to identify the optimal lymphoconditioning strategy. Given the success seen in the above trials, combinations of NK cells with ICIs deserve further study. Another consideration is that traditional NK cell therapies like the ones used in the trials above have no specific antigen target. However, an advantage of NK cells is their ability to easily be paired with bispecific killer engagers (BiKEs) or trispecific killer engagers (TriKEs), which can enhance NK-cell mediated immunity by linking target cells with NK cells through engagement of CD16 and triggering ADCC [18,36]. Miller et al. (2022) tested a TriKE that consisted of an IL-15 component, anti-CD16 component, and an anti-MSLN component and found that the TriKE improved NK cell activity in hypoxic conditions in vitro against NSCLC cell lines [144]. Several other preclinical studies have shown beneficial effects when NK cells are combined with BiKEs or TriKEs [145,146,147,148,149,150,151]. Another approach to direct NK cells to target cells was explored by Snyder et al. (2023) [152]. They created NK cells derived from iPSCs which expressed both an IL-15/IL-15 receptor α fusion protein and a fusion protein composed of the extracellular portion of CD64 fused to the transmembrane and intracellular signaling domains of CD16a. The addition of tumor antigen-specific monoclonal antibodies led to enhanced ADCC via the CD64/16A fusion protein and allowed for efficient tumor antigen targeting in ovarian cancer cell lines and in mice with ovarian cancer xenografts. These strategies should be explored in future clinical trials to enhance NK cell tumor targeting.

A summary of published clinical trials of TILs, CAR-T cells, TCR-T cells, and NK cells in patients with thoracic malignancies is provided in Table 2.

## 12. Barriers to Success of Cellular Immunotherapies

**Figure 1:**
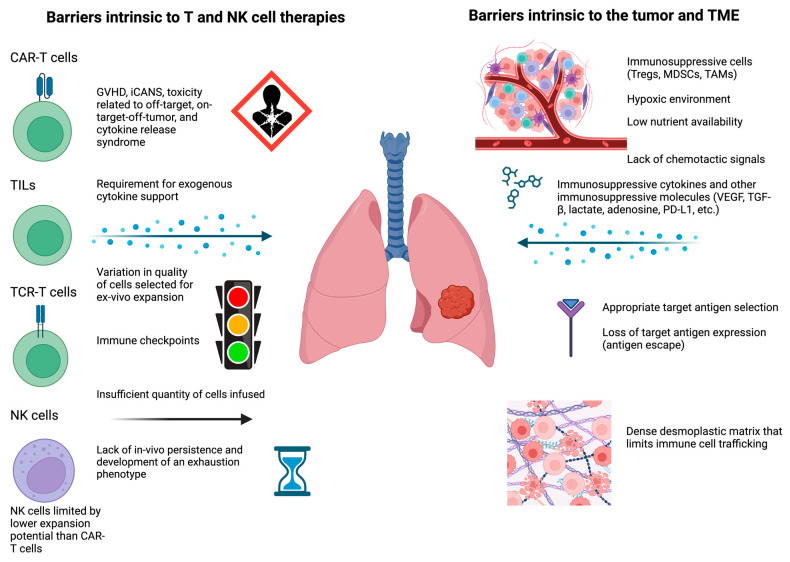
Overview of the barriers faced by cell therapies in the treatment of thoracic cancers. Tumor and TME-intrinsic factors limiting the efficacy of cell therapies include selection of an appropriate antigen target, antigen escape, hypoxia, immunosuppressive cytokines and chemicals such as lactate and adenosine, low nutrient availability, lack of chemotactic signals to promote immune cell recruitment, immunosuppressive cells (Tregs, MDSCs, TAMs, etc.), and a dense desmoplastic matrix that prevents effective trafficking of immune cells to tumor cells. Limitations intrinsic to T and NK cell therapies include an insufficient quantity of cells delivered to the tumor, variation in the quality of cells selected for ex vivo expansion, inability to traffic through the TME to tumor cells, lack of persistence, development of an exhausted phenotype, requirement of exogenous cytokine support, the presence of immune checkpoints, the risk of GVHD, iCANS, and toxicity related to off-target, on-target, off-tumor, and cytokine release syndrome. These limitations intrinsic to the cell therapies are shared amongst each of the cell therapy types discussed. Created in BioRender. Erickson, S. https://BioRender.com/p99g888 (accessed on 16 December 2024).

**Figure 2:**
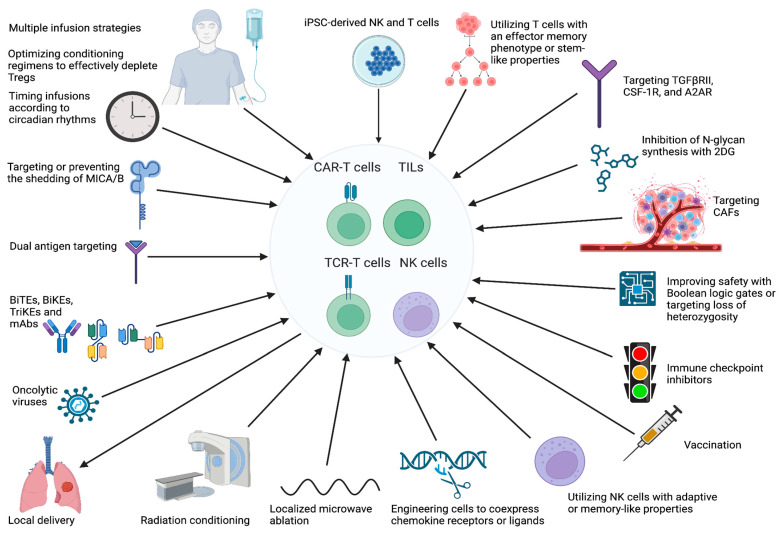
Strategies being explored to improve the efficacy of cell therapies in thoracic cancers. Cell source is an important consideration for cell therapy products. iPSCs allow for efficient expansion of multigene edited off-the-shelf T and NK cell therapies and are a promising avenue to explore in future clinical trials. Additional considerations that may optimize the efficacy of T and NK cell therapies include: repeat infusion strategies to overcome the issues of persistence and insufficient quantity of cells; optimizing conditioning regimens to effectively deplete Tregs; timing infusions according to circadian rhythms present in T cells and endothelial cells in the TME; dual antigen targeting to prevent antigen escape; combination with BiTEs, BiKEs, TriKEs, or mAbs to improve antitumor efficacy via improved targeting and improve safety by increasing specificity; combination with oncolytic viruses; combination with vaccines; targeting or preventing the shedding of MICA/B; sensitization with radiation conditioning; improving CAR-T cell trafficking with localized microwave ablation; targeting CAFs which produce the dense desmoplastic matrix of the TME; engineering cells to co-express chemokine receptors or ligands; inhibiting N-glycan synthesis with 2DG; blocking CSF-1R with antibodies, genetic deletion or inactivation of TGFβRII, and genetic deletion of A2AR; local delivery of CAR-T cells to bypass the issue of effective trafficking to target sites; utilizing T cells with an effector memory phenotype or stem-like properties; and utilizing NK cells with adaptive or memory-like properties. Created in BioRender. Erickson, S. (2024). https://BioRender.com/a73j495 (accessed on 29 October 2024).

A major limitation to the success of CAR-T cell products in solid tumors is the challenge of delivering a sufficient number of CAR-T cells to the tumor [11]. This is due to a multitude of factors present in the immunosuppressive tumor microenvironment (TME), including suppressive cells, suppressive cytokines, hypoxia, and other physical and chemical barriers [11]. TCR-T cells, TILs, and NK cells face essentially the same obstacles [5,17,18]. Identification of ideal antigen targets is another major barrier [5,16,17,18]. Several ongoing early-stage clinical trials are utilizing recent basic science advances to target these barriers with the goal of advancing a product to the clinic (Table 3). Figure 1 provides an overview of the major obstacles that must be overcome for T and NK cell-based cellular immunotherapies to prove effective in thoracic cancers. Strategies to address these barriers are summarized in Figure 2.

## 13. Antigen Escape

Besides the challenge of finding an ideal antigen target, one of the key obstacles faced by CAR-T cell therapies is the development of resistance secondary to loss of target antigen expression [16]. To combat this, Wei et al. (2017) utilized anti-PSCA and anti-MUC1 CAR-T cells and found synergistic tumor killing ability when used in combination in a mouse model of NSCLC [44]. Numerous other preclinical studies have shown a benefit in solid tumor models when multiple antigens are targeted [21,153,154]. Several clinical trials currently enrolling have leveraged a multi-antigen targeting approach and are using CAR-T cells targeting multiple antigens or combinations of CAR-T cells each targeting a single antigen: CAR-T cells targeting EGFR and B7-H3 (NCT05341492), CAR-T cells targeting GPC3 and transforming growth factor β (TGFβ) [155] (NCT03198546), and CAR-T cells directed against one of a multitude of targets to be given alone or in combination (NCT03198052). Another approach which may be worth exploring is combining CAR-T cells with bispecific T cell engagers (BiTEs). Choi et al. (2019) developed a CAR-T cell with an anti-EGFRvIII CAR that also expressed an anti-EGFR BiTE and tested its antitumor activity in mouse models of glioblastoma [156]. The authors found that these CAR-T cells could more effectively control growth of tumors with variable levels of EGFRvIII expression compared to an anti-EGFRvIII CAR that did not secrete a BiTE and were also able to recruit bystander T cells. Another approach to combat antigen escape is by inducing epitope spreading. Lai and colleagues (2020) engineered both CAR-T cells and TCR-T cells to secrete Fms-like tyrosine kinase 3 ligand (FLT3L), a dendritic cell growth factor [157]. When combined with immune adjuvants, these FLT3L-secreting T cells promoted conventional type 1 dendritic cell (cDC1) proliferation and subsequent T cell epitope spreading and resulted in tumor regression in mice with solid tumors. Another method employed by tumors to evade immune detection is aberrant glycosylation of potential target proteins, which functionally hides antigens from the immune system by impairing formation of an immune synapse. Greco et al. (2022) sought to target this by using 2-deoxy-D-glucose (2DG) to inhibit N-glycan synthesis. They found that treatment with 2DG improved CAR-T cell killing capacity against lung cancer cell lines and cell lines of several other solid tumors, inhibited PD1/PD-L1 interactions, and prevented the development of an exhaustion phenotype [158]. Toyofuku et al. (2024) took a similar approach by transducing an anti-FMR1NB CAR into T cells that had been pretreated with 2DG and found these CAR-T cells had improved cytotoxicity against a mouse model of lung cancer [29]. They found that 2DG treatment of T cells suppressed N-glycosylation of T cells and led to reduced binding of the immunosuppressive ligands PD-L1, CD86, and galectin. This strategy is interesting, as it could be easily incorporated into CAR-T cell therapy treatment protocols. Another strategy to combat the issue of antigen-negative escape variants explored by DeSelm et al. (2018) used radiation conditioning directed at the tumor [159]. They tested this approach in a mouse model of pancreatic cancer that had variable expression of the target antigen sialyl Lewis-A and found that low-dose radiation conditioning sensitized antigen-negative tumor cells to TRAIL-mediated cell death by the CAR-T cells, which were activated to produce TRAIL after encountering antigen-positive tumor cells. Importantly, radiation conditioning did not induce expression of the target antigen. NCT05576077 is investigating autologous TILs in combination with pembrolizumab, IL-2, and low-dose radiation therapy in patients with advanced solid tumors.

Another concept worth further investigation is the idea of targeting tumor cells in ways other than traditional antigen targets like those described in Table 1. For example, major histocompatibility complex (MHC) class I polypeptide-related sequence A (MICA) and B (MICB) are stress-related proteins that are often upregulated on tumor cells but not healthy cells and can be recognized by NK group 2 member D (NKG2D) receptors expressed on CD8+ T cells and NK cells, as well as several other NK cell receptors [34,36]. Tumor cells can evade immune detection by shedding MICA/B via proteolytic cleavage at the α3 domain of MICA/B [34]. To target this, Goulding and colleagues (2023) developed iPSC-derived NK cells which expressed a CAR directed against the conserved MICA/B α3 domain and found that these cells had antitumor activity against an NSCLC cell line [34]. Another unique approach to targeting tumor cells that may circumvent the issue of target antigen loss is the concept of loss of heterozygosity [37]. This clonal loss of an allele is irreversible, common in many cancers, and highly specific to cancer cells, which makes it an ideal way to specifically target cancer cells and has been identified as a possible contributor to immune evasion in NSCLC [37,38]. With this concept in mind, Hwang et al. (2021) designed a CAR-T cell with a “NOT-gate” in which T cells were engineered to express two CARs [37]. One CAR was directed against the human leukocyte antigen (HLA) allele still present in the tumor cells and the other was an inhibitory CAR (iCAR) directed against the HLA allele which the tumor had lost expression of. They termed this design “neoplasm-targeting allele-sensing CAR” (NASCAR), and these NASCAR T cells specifically identified tumor cells and induced cytotoxic responses against the lung cancer cell line NCI-H441 in vitro.

## 14. Delivering Cells to the Tumor

Another major obstacle is delivering CAR-T cells to the tumor cells in the immunosuppressive TME [11]. One approach that has been explored to address the many obstacles faced by CAR-T cells in trafficking to the tumor cells in the TME is bypassing part of this process with local delivery of CAR-T cells. Smith et al. (2017) tested this concept by developing a biopolymer scaffold that delivered CAR-T cells and cyclic di-GMP, a stimulator of interferon genes (STING) agonist, directly at the tumor site to efficiently mediate tumor regression and induce a broader immune response in mouse models of solid tumors [160]. Adusumilli et al. (2014) also leveraged this concept in a mouse model of MPM and found that intraperitoneal delivery of MSLN-directed CAR-T cells resulted in superior antitumor activity and functional persistence compared to intravenous delivery of CAR-T cells [161]. Klampatsa et al. (2017) developed panErbB (EGFR (ErbB1), HER2 (ErbB2), ErbB3, and ErbB4)-directed CAR-T cells which also expressed IL-4 receptor to allow for CAR-T cell proliferation and found that intraperitoneal injection of these CAR-T cells mediated tumor regression in a mouse model of MPM [33]. The trials by Hiltbrunner et al. (2021) and Adusumilli (2021), which utilized intrapleural administration of anti-FAP CAR-T cells and anti-MSLN CAR-T cells, respectively, showed hints of efficacy and a good safety profile in patients with MPM, and this strategy should continue to be explored [118,119]. As MPM is a local disease, this strategy has great promise and should continue to be explored in future trials.

## 15. Improving Cell Trafficking to Tumors

Another approach to help CAR-T cells reach the TME utilizes chemokines or other signaling molecules. The chemokines expressed in the TME in solid tumors favor the recruitment of suppressive cells and hamper the recruitment of desired immune cells that can control tumor growth [155]. Several preclinical studies have shown improved CAR-T cell trafficking to target locations when CAR-T cells are engineered to co-express a chemokine receptor or ligand [162,163,164,165]. Shi et al. (2024) took an innovative approach to address the issue of delivery of CAR-T cells to the TME [166]. They loaded CAR-T cells into human lyophilized lymph nodes and found that implantation of these CAR-T cell-loaded lymph nodes in mice with solid tumor xenografts reduced tumor recurrence compared to a hydrogel delivery system supplemented with supporting cytokines. This approach allowed for a more physiological environment for CAR-T cells with optimal exposure to chemokines, cytokines, and other stimulatory molecules to promote CAR-T cell function. Cao et al. (2022) took another unique approach to improve CAR-T cell infiltration [41]. They tested anti-AXL CAR-T cells in mice with AXL+ NSCLC xenografts and found that combining CAR-T cells with local microwave ablation led to improved CAR-T cell infiltration, improved tumor regression, greater partial pressure of oxygen in the TME, and improved oxidative metabolism in these CAR-T cells compared to CAR-T cell monotherapy. This approach was safe in their preclinical models and should be explored in human trials. An additional challenge with trafficking CAR-T cells through the TME is a dense desmoplastic matrix surrounding and protecting tumor cells. One type of stromal cell present in the TME, cancer-associated fibroblasts (CAFs) are one of the cell types responsible for production of this matrix and have thus been identified as a target to improve the efficacy of cell therapies [155,167]. Xia et al. (2023) aimed to target this by using CAR-T cells directed against FAP, which has high levels of expression on CAFs, in a mouse model of pancreatic cancer followed by anti-MSLN CAR-T cells [167]. The anti-FAP CAR-T cells effectively eliminated stromal cells and depleted the immunosuppressive matrix, and subsequent treatment with anti-MSLN CAR-T cells led to improved tumor control, suggesting that stromal depletion sensitized the tumor to treatment with the anti-MSLN CAR-T cells. Das et al. (2023) observed similar results with sequential treatment with anti-FAP CAR-T cells followed by anti-MSLN CAR-T cells in a mouse model of triple-negative breast cancer [168]. Engineering CAR-T cells to express heparanase is an additional strategy that may effectively degrade the dense extracellular matrix (ECM) and promote CAR-T cell infiltration [169]. Liu et al. (2021) tested anti-B7-H3 CAR-T cells as well as a bispecific killer cell engager (BiKE) directed against B7-H3 in vitro and in a mouse model of NSCLC. They found that anti-B7-H3 CAR-T expression in T cells promoted tumor infiltration compared to control T cells. They also found that anti-B7-H3 BiKE led to improved NK cell activation and tumor control [145]. It should also be noted that, besides the chemokines and other signaling molecules mentioned above, tumor expression of HLA class I and PD-L1 are positively and negatively correlated with intratumoral T cell infiltration, respectively [170,171].

## 16. Targeting Immunosuppressive Cytokines and Other Signaling Molecules

TGFβ is a suppressive cytokine that promotes several changes in the TME that favor tumor growth and resist antitumor immune cell infiltration, such as increasing production of collagen and other ECM proteins in the TME, which leads to a dense ECM that impedes T cell infiltration and also by promoting an immunosuppressive phenotype in stromal cells [155]. Several studies have investigated methods to abrogate the negative effects of TGFβ on CAR-T cell function. Kloss et al. (2018) designed anti-PSMA CAR-T cells to express a dominant negative TGFβ-receptor II (TGFβRII) and investigated the activity of these cells against a prostate cancer cell line and in a mouse model of prostate cancer [172]. The authors found that these cells exhibited enhanced antitumor activity and proliferative capacity compared to anti-PSMA CAR-T cells with intact TGFβR2 function. Tang and colleagues (2020) took a similar approach by constructing TGFβRII knockout (KO) CAR-T cells and found that these edited CAR-T cells had superior antitumor activity in pancreatic cancer models [173]. They also created double KO CAR-T cells in which both PD-1 and TGFβRII were knocked out, which led to even greater antitumor efficacy. Lane and colleagues (2024) developed a method of targeting endogenous proteins by constructing bifunctional degrader proteins that linked an E3 ligase to SMAD2/3, downstream transcription factors involved in TGFβ signaling [174]. This strategy ultimately results in ubiquitination of the target protein and subsequent degradation. In their study, this complex improved in vivo CAR-T cell antitumor activity and proliferative capacity. This method is also unique, as it can potentially be repurposed to target additional target proteins of interest. As the authors note, targeting this downstream pathway may avoid resistance mechanisms that tumor cells may employ when TGFβ is targeted at the receptor level. Colony stimulating factor 1 (CSF-1) is another immunosuppressive cytokine present in the TME that mediates its suppressive effect by recruiting suppressive myeloid cells and promoting macrophage differentiation toward an M2 phenotype and preclinical studies suggest targeting its receptor, CSF-1 receptor, may be an effective way to increase T cell infiltration and improve the ability of T cell-based therapies to control tumor growth in solid tumors [175,176]. Adenosine, an immunosuppressive molecule derived from ATP and produced via sequential action of the ectonucleotidases CD39 and CD73, tends to be present in high concentrations in the TME and is known to impair antitumor responses in lymphocytes and contribute to solid tumor progression [177]. Targeting the action of adenosine at the adenosine 2A receptor (A2AR) results in improved CAR-T cell function [178,179]. Additional immunosuppressive cytokines and molecules present in the TME include IL-4, IL-6, IL-10, vascular endothelial growth factor (VEGF), and lactate, and various strategies have been explored to counteract their effects [16,155]. Somewhat counterintuitively, however, Zhao et al. (2024) developed a CAR-T cell which co-expressed IL-10 and observed increased oxidative phosphorylation, proliferation, and antitumor activity in a mouse model of pancreatic cancer [180].

## 17. Cytokine Support

One approach that has been explored to overcome the immunosuppressive TME is the use of cytokines in combination with CAR-T cells. Multiple preclinical studies have shown that engineering CAR-T cells to express proinflammatory cytokines in various combinations (IL-2 and IL-33 [181], IL-12 [182], IL-15 [183], IL-15 and IL-21 alone or in combination [184], IL-18 [59,185,186], and IL-23 [187]) leads to improved CAR-T cell proliferation and control of tumor growth in mouse models of solid tumors. Allen et al. (2022) designed a CAR-T cell that also expressed a synthetic notch receptor to create an autocrine IL-2 circuit [188]. This approach led to improved antitumor activity and CAR-T cell infiltration in mouse models of solid tumors. Notably, this avoided systemic toxicity that can be seen with systemic administration of IL-2. Another strategy that tumors can use to combat an immune response is secretion of small extracellular vesicles (sEv) that contain immune checkpoint ligands and tumor antigens which lead to suppressed CAR-T cell function. Zhong et al. (2023) found that CAR-T cell treatment leads to greater production of PD-L1 on sEV released by tumor cells and that this negatively affects CAR-T cell infiltration, proliferation, and antitumor activity [189]. Remarkably, they found that these functions can be rescued by co-administration of CAR-T cells with an exosome inhibitor in a murine solid tumor model [189].

## 18. Targeting Immunosuppressive Cells

Multiple cell types in the TME are immunosuppressive and inhibit CAR-T cell efficacy. One such cell type is regulatory T cells (Tregs), which mediate their immunosuppressive effect through secretion of TGFβ and by acting as cytokine “sinks”, and their presence is negatively associated with response to TIL therapy [155,190]. In an effort to selectively deplete Tregs, Zhang et al. (2021) developed a heterodimeric molecule directed against CTLA-4 and signal regulatory protein α (SIRPα), a ligand of CD47 [191]. This effectively blocked the “do not eat me” signal of CD47 and allowed for selective Treg depletion in murine models of colon cancer [191]. Similar to T cells, Tregs also impair NK cell function [18]. Bachanova et al. administered allogeneic NK cells to patients with acute myeloid leukemia with or without an IL-2 diphtheria toxin fusion protein, which effectively depleted Tregs [192]. The patients treated with IL-2 diphtheria toxin fusion protein also had improved rates of complete remission at day 28 and OS at 6 months. Of note, depletion of Tregs is thought to be one of the mechanisms by which lymphodepletion improves the efficacy of ACT [193]. The trials reported in Table 2 largely used some combination of cyclophosphamide and fludarabine for lymphodepletion. Addition of oxaliplatin to standard lymphodepleting chemotherapy regimens should also be explored, as addition of oxaliplatin to cyclophosphamide in a murine model of lung cancer was shown to promote CAR-T cell recruitment to tumors via stimulation of chemokine secretion by macrophages and also improved antitumor immune responses when paired with anti-PD-L1 antibodies [194].

Additional cells present in the TME include tumor-associated macrophages (TAMs), which secrete immunosuppressive cytokines and increase PDL1 expression and myeloid-derived suppressor cells (MDSCs), which act through a similar mechanism [155]. MDSCs are a diverse group of myeloid cells that prevent effective antitumor immune responses and several methods of targeting these cells have been investigated in preclinical studies. Luo et al. (2022) used a folate-targeted Toll-like receptor 7 agonist to effectively and specifically target MDSCs and TAMs which promoted their differentiation from a pro-tumor/anti-inflammatory M2 phenotype toward an antitumor M1 phenotype [195]. Srivastava et al. (2012) used a murine model of lung cancer and found that using antibodies to deplete MDSCs enhanced antitumor immune responses [196]. Johnson et al. (2021) engineered CAR-T cells to co-express an immunostimulatory RNA molecule and found that this impaired MDSC development, decreased TGFβ production by myeloid cells, and led to enhanced CAR-T cell proliferation and tumor control in mice with solid tumors [197].

## 19. Metabolism, Exhaustion and Persistence

Additional barriers to success of CAR-T cells include a lack of persistence of functional CAR-T cells over time and the development of an exhausted phenotype after an initial period of functional cytotoxicity [11]. Prolonged or tonic stimulation leads to epigenetic and transcriptional changes in T cells that result in an exhausted phenotype with impaired effector functions, which is a major obstacle limiting the effectiveness of CAR-T cell therapies and numerous approaches have been explored to prevent or overcome exhaustion [16,155]. Mitochondrial dysfunction is a key feature of exhaustion in T cells and is thus a target for improving the efficacy of T cell-based therapies. In a first-of-a-kind approach, Baldwin et al. (2024) utilized intercellular transfer of mitochondria derived from bone marrow stromal cells through nanotubes into CD8+ T cells to improve the metabolic fitness of the T cells [198]. T cells treated with this mitochondrial transfer strategy had better antitumor efficacy and further enhanced survival in mice with solid tumors compared to mice treated with T cells and no mitochondrial transfer. Another strategy that could be incorporated into multigene edited cell therapy products using CRISPR-Cas13d or other multiplexed genome editing approaches is the deletion of sorting nexin 9 (SNX9), as knockout of this gene has been shown to prevent exhaustion and promote a memory phenotype in T cells [89,199].

In order to address the issue of limited persistence of CAR-T cells, an innovative approach that could be considered is simply using multiple infusions of CAR-T cells over time to circumvent the issues of persistence and exhaustion [11]. While this poses concerns about the cost of multiple infusions, novel techniques for expansion of CAR-T cells and the prospect of iPSC-derived products may help to cut costs in the near future and make this approach feasible and should be explored [4]. An additional approach to improve CAR-T cell persistence was explored by Garcia and colleagues (2024) and involves exploiting a gene fusion found naturally in T cell lymphomas [200]. T cells engineered to express this gene fusion, *CARD11-PIK3R3*, had improved persistence and tumor control in mice with solid tumors. Another unique approach that could be explored is optimizing the timing of CAR-T cell administration. Recent work by Wang et al. (2024) revealed that circadian rhythms operating in both endothelial cells of the TME and T cells impact tumor infiltration and antitumor responses and that optimal timing of CAR-T cell infusions resulted in improved tumor control in a mouse model of diffuse large B cell lymphoma [201].

Another consideration that has been raised is that many trials of CAR-T cells have used T cells with either a naive or central memory-like phenotype that favors lymph node and bone marrow migration and may partially explain why CAR-T cells have seen success in hematologic malignancies but not solid tumors [11]. Therefore, using CAR-T cells which have either an effector or effector memory phenotype that traffic to the peripheral sites required for efficacy in solid tumors may be a better approach in thoracic malignancies and other solid tumors [11]. A related consideration is the observation that a population of CD8+ stem-like T cells seem to be key to an effective antitumor response in T cell-based ACT in solid tumors [202] and expression of the transcription factor FOXO1 seems to be crucial for CAR-T stemness and memory properties [203,204]. Wang et al. (2024) recently reported a method of developing CAR-T cells with these desirable stem-like and memory properties via repression of two proteins involved in transcriptional regulation, ZC3H12A and BCOR [205]. With a similar goal in mind, Sukumar et al. (2024) developed a method for sorting T cells according to their mitochondrial membrane potential and found that T cells with a lower mitochondrial membrane potential had superior metabolic fitness and stem-like and memory phenotypic properties [206]. These T cells exhibited superior persistence and tumor control in mice with solid tumors. Future trials should consider using these or related techniques to ensure CAR-T cells have these favorable phenotypic properties. Lastly, engineered immune cells face a lack of necessary nutrients in the TME, and novel strategies are also needed to address this obstacle [91,155].

## 20. Targeting Tumor Hypoxia

Hypoxia is another feature of the TME that contributes to immunosuppression and has been shown to inhibit CAR-T cell function in vitro [155,207]. Several groups have designed CAR-T cells which function only under hypoxic conditions, which increases safety and specificity of CAR-T cell action and may also favorably impact CAR-T cell metabolic fitness and improve the efficacy of CAR-T cell treatment [208,209,210].

## 21. Targeting Immune Checkpoints

Multiple immune checkpoints exist in T cells which act to maintain tolerance to self in healthy individuals but can be upregulated through various mechanisms in the setting of malignancy, which promotes exhaustion and impairs T cell effector function, contributing to the failure of ACT [16,211]. Several strategies to disrupt the checkpoints PD-1 [45,125,163,212] and cytotoxic T lymphocyte antigen 4 (CTLA-4) [213] have been explored, including anti-checkpoint scFv, blocking antibodies, and genetic disruption. Manipulation of these checkpoints in these preclinical studies suggests that this strategy may be an effective method of improving antitumor efficacy and preventing or reversing exhaustion in CAR-T cells. The greatest success has been seen with methods that disrupt the PD-1/PD-L1 axis, but additional immune checkpoints implicated in control of T cell function that should be explored in the context of CAR-T cell therapy include V-domain immunoglobulin suppressor of T cell activation (VISTA), T cell immunoreceptor tyrosine-based inhibitory motif domains (TIGIT), TIM-3, lymphocyte activation gene 3 (LAG3), and B7-H3 [211]. Results from the preclinical studies mentioned suggest targeting one or several of these checkpoints with either blocking antibodies or genetic disruption could be easily implemented into a CAR-T cell product with multiple other genetic alterations designed to enhance CAR-T function. Immune checkpoint inhibition may also be combined with TIL therapy and *CISH*, a checkpoint that mediates its suppressive effect through disruption of TCR signaling, may be an ideal target [214]. Palmer and colleagues (2020) found that *CISH* KO in TILs leads to improved proliferation and recognition of neoantigens, and that administering these genetically engineered TILs in combination with inhibition of PD1 led to improved antitumor responses [215]. These *CISH* KO TILs are currently under investigation in a phase 1 trial for patients with advanced gastrointestinal tumors (NCT04426669).

## 22. Combination with Oncolytic Viruses

Another approach worth considering is combination therapy with oncolytic viruses, which are engineered viruses that selectively infect malignant cells and have the potential to favorably impact the immune response against tumors through various mechanisms [216]. Multiple preclinical studies suggest combination therapy with oncolytic viruses may enhance CAR-T cell efficacy [217,218,219,220,221]. NCT03740256 is investigating anti-HER2 CAR-T cells in combination with intratumoral injection of an oncolytic adenovirus in patients with advanced or refractory HER2-positive solid tumors.

## 23. Combination with Vaccines

A novel strategy of “vaccine-boosting” was explored by Ma and colleagues (2019). They tested an amphiphile CAR-T ligand in mice with solid tumors and found that this ligand traveled to lymph nodes and was able to promote CAR-T cell proliferation and infiltration, improve tumor control, and increased CAR-T cell polyfunctionality [222]. Further research by the same group revealed that this “vaccine-boosting” strategy can promote antigen spreading and effectively eliminated solid tumors with heterogeneous target antigen expression in mouse models of solid tumors [223]. A similar promising strategy was explored by Reinhard et al. (2020) [224]. They used a lipoplex RNA vaccine to induce claudin 6 (CLDN6) expression on dendritic cells in combination with CAR-T cells directed against CLDN6 and found that this strategy improved in vivo CAR-T cell expansion and antitumor responses in mouse models of solid tumors. This strategy is already being explored in phase 1 clinical trials: NCT04503278 is investigating CAR-T cells directed against CLDN6 in patients with advanced CLDN6-expressing solid tumors to be given alone or in combination with an RNA vaccine contained within lipoplexes; and NCT06253520 is investigating TCR-T cells specific for KRAS G12V or G12D mutations in patients with advanced solid tumors to be given with a vaccine against these same targets. Thus, combining T cell-based cellular immunotherapies with vaccination is a promising strategy to augment their effectiveness.

## 24. Future Directions for TIL Therapy

Attempts to identify biomarkers associated with ideal TILs have identified CD39 and CD107 co-expression, as well as CD137 expression on T cells to be associated with tumor-reactive TILs [225,226]. Several groups have also utilized single-cell RNA sequencing to identify gene expression patterns characteristic of neoantigen-reactive T cells, which could prove useful in the development of future generations of TILs [112,227,228]. Transcriptomics has also been successfully leveraged to identify TILs capable of inducing clinical responses [229]. In addition, while identification of TILs reactive against specific neoantigens is important, polyclonal or multi-specific TILs are likely to be more effective due to eventual development of antigen-negative clones [230,231,232]. In fact, Dolton et al. (2023) analyzed TILs from a patient with metastatic melanoma who had a remarkable response to TIL therapy and found the dominant clonotype, present in the patient over a decade after complete remission, recognized multiple epitopes derived from several unique tumor-associated antigens in cell lines of several cancers [233]. The authors suggest this finding implies the TILs did not target a single unique neoepitope, but rather several common shared antigens. These results further reinforce the idea that targeting a single epitope is insufficient for achieving the goal of inducing long term responses. However, a large number of TCRs may not be required, as Wolf et al. (2024) found that a combination of a single neoantigen-specific CD4+ TCR with a single neoantigen-specific CD8+ TCR was necessary and sufficient to effectively eliminate solid tumors, while multiple neoantigen-specific CD8+ TCRs was associated with relapse in heterogeneous tumors [234]. Novel approaches such as immunomagnetic cell sorting to increase TIL expansion and in vitro stimulation with neoantigens to selectively expand neoantigen-reactive TILs with a stem-like phenotype are also promising strategies that could improve the efficacy of TIL therapy [235,236].

## 25. Future Directions for CAR-T Cell Therapy

Preventing toxicity is crucial to the success of any CAR-T cell product and is a major focus of ongoing research. Besides appropriate antigen selection, several other methods are being investigated to improve the safety of CAR-T cells. Tokatlian et al. (2022) constructed MSLN-directed CAR-T cells that also expressed an inhibitory receptor specific for HLA-A*02 [237]. This allowed the CAR-T cells to detect tumor cells with loss of heterozygosity at HLA-A*02 and avoid activation in healthy tissue. Another method to ensure CAR-T cells are active only against tumor cells was explored by Srivastava and colleagues (2019) [43]. They designed ROR1-directed CAR-T cells to co-express EpCAM or B7-H3-specific synthetic notch receptors which functioned as a logic gate to restrict CAR-T cell activity to tumor cells that expressed the ligands for the synthetic notch receptors. Chu et al. (2020) developed ligand-controlled CAR-T cells directed against a linker molecule composed of fluorescein isothiocyanate conjugated to folate (folate-FITC) that was specific for both FRα and folate receptor-β (FRβ) [238]. These CAR-T cells were thus capable of targeting both lung cancer cells expressing FRα and TAMs expressing FRβ and CAR-T cell activity required the presence of folate-FITC, improving their safety and specificity. Numerous other strategies to improve the safety of CAR-T cells have been explored (bispecific CARs, various iterations of Boolean logic gates, suicide genes/receptors, drug/antibody-inducible activation platforms, etc.) and are reviewed in detail elsewhere [16,155]. One last exciting new technology in preclinical stages of development that we will mention is that of in vivo generation of CAR-T cells, which can be accomplished using viral vectors or enveloped particles containing CRISPR-Cas9 and a guide RNA. Besides potential savings in cost and manufacturing time, this technology has the benefit of avoiding the need for ex vivo engineering and re-infusion of CAR-T cells, as well as no requirement for lymphodepletion [239,240,241].

## 26. Future Directions for TCR-T Cell Therapy

As described above, results from trials of TCR-T cells in thoracic cancers have been unsuccessful to date and safety remains a concern for TCR-T cells [17]. However, there is reason to remain optimistic that TCR-T cells could one day prove successful, as there have been examples of clinical responses in patients with advanced solid tumors. Tran et al. (2016) isolated TILs from a patient with metastatic colorectal cancer bearing a KRAS G12D mutation [242]. These TILs were expanded ex vivo prior to reinfusion, and the patient was noted to have a PR characterized by regression of their pulmonary metastases. Characterization of these TILs revealed four clonotypes, each of which were HLA-C*08:02-restricted. Analysis of a tumor sample upon progression at 9 months revealed loss of HLA-C*08:02 expression. In a follow-up report by the same group, Leidner et al. (2022) treated a patient with metastatic pancreatic adenocarcinoma with a KRAS G12D mutation with HLA-C*08:02-restricted autologous T cells transduced to express two allogeneic TCRs reactive against this KRAS G12D mutation [243]. A PR characterized by regression of pulmonary metastases was ongoing at a 6-month follow-up.

In an effort to address safety concerns, Foldvari et al. (2023) developed a method to comprehensively screen candidate TCRs for cross-reactivity [244]. Careful selection of TCRs reactive against patient-specific neoepitopes is another major challenge. Recent advances in our understanding of ideal physical features of candidate epitopes and computer modeling of peptide–MHC binding interactions will aid in the selection of appropriate neoantigen-specific TCRs that promote antitumor immunity while minimizing the potential of cross-reactivity [245,246,247,248]. Furthermore, Ding et al. (2023) developed anti-MSLN CAR-T cells and TRuC-T cells specific for MSLN and found that the TRuC-T cells exhibited more rapid killing of target cells in vitro and had more favorable metabolic characteristics, with greater mitochondrial metabolism and lower levels of glycolysis than the CAR-T cells [249]. Thus, despite safety concerns, TCR-T cells warrant further investigation.

## 27. Future Directions for NK Cell Therapy

NK cells face the same barriers in the TME as CAR-T cells and other cellular therapies, and similar strategies have been employed to combat this [18,36]. For instance, NK cells have many of the same checkpoints as T cells and inhibiting these checkpoint pathways with monoclonal antibodies or genetic manipulation has also proved beneficial for NK cell-mediated tumor immunity [18,36,250]. These checkpoints include PD-1, CISH, TIGIT, NKG2A, inhibitory KIRs, LAG3, TIM-3, and others [18,250,251,252,253,254,255,256]. Agonists of several NK cell activating receptors have also been explored preclinically [18,250]. As described previously, targeting MICA/B with CAR-NK cells is an effective way to specifically target solid tumors [34]. Improved NK cell-mediated antitumor responses can also be accomplished by preventing the shedding of these proteins with antibodies or increasing their expression with the histone deacetylase inhibitor panobinostat [257,258]. Reggiani et al. (2024) sought to target multiple NK cell inhibitory pathways simultaneously [259]. They used Bromodomain and Extra-Terminal protein inhibitors (BETi) to induce broad epigenetic changes in NK cells that led to decreased expression of inhibitory KIRs and several immune checkpoint proteins, such as PD1, CLTA-4, LAG3, and several others. Treatment with BETi improved NK cell cytotoxicity against NSCLC cell lines and led to greater antitumor responses in mice with NSCLC xenografts treated with adoptively transferred NK cells. Another unique approach to enhance NK cell efficacy focuses on improving the ability of NK cells to efficiently trigger apoptosis in target cells, as resisting mitochondrial apoptosis is a mechanism of resistance to NK cell therapies. Pan et al. (2022) used BH3 mimetics to enhance NK cell antitumor activity by lowering the threshold for triggering mitochondrial apoptosis in target cells [260]. They observed improved NK cell-mediated tumor control in vitro and in a mouse model of cervical cancer, making using BH3 mimetics a promising strategy to explore in clinical trials of NK cell therapy.

Similar to T cells, NK cells can also be engineered to express a CAR or TCR, and several preclinical studies have found CAR-NK cells or TCR-NK cells to have potent antitumor activity [261,262,263,264,265]. Combination strategies with CAR-T cells may also be worth considering, as Parihar et al. (2019) developed an NK cell that expressed a chimeric receptor consisting of an activating NKG2D receptor and the ζ-chain of a TCR and found that these modified NK cells selectively eliminated MDSCs and allowed for improved antitumor responses and infiltration of anti-GD2 CAR-T cells in a mouse model of neuroblastoma [266].

A potential drawback of NK cells as compared to T cell-based therapies is a lack of functional persistence; therefore, strategies such as repeat infusions and local delivery should also be explored for NK cell-based therapies [11,18]. An additional way to circumvent this lack of functional persistence could be to utilize special populations of NK cells. Cooper et al. (2009) stimulated NK cells with IL-12, IL-15, and IL-18 and found that this conferred memory-like properties [267]. These cytokine-induced memory-like (CIML)-NK cells had an enhanced ability to produce IFN-γ upon restimulation. Early phase clinical trials have shown these CIML-NK cells can mediate tumor regression in patients with acute myeloid leukemia and head and neck squamous cell carcinoma [268,269]. Another unique type of NK cells with favorable functional characteristics, termed adaptive NK cells, are a population of NK cells that form upon exposure to cytomegalovirus infection [270,271]. They are characterized by CD57 and NKG2C expression, silencing of the transcription factor PLZF, and acquisition of a DNA methylation pattern similar to cytotoxic T cells [270,271]. Preclinical studies have also shown that these adaptive NK cells may be inherently resistant to MDSCs and Tregs [272,273]. Woan et al. (2021) developed iPSC-derived NK cells with functional properties similar to adaptive NK cells, which could make this approach more feasible for clinical translation of a uniform adaptive NK cell therapy product [274]. In recent years, gene-edited iPSCs and methods for NK cell differentiation have advanced rapidly. Clinical trials for relapsed/refractory diseases are promising with these new technologies. We expect platforms to improve and clinical trials to reach late stages and commercialization. Next-generation immunotherapies using banked allogeneic iPSC-derived NK cells are within reach.

## 28. Conclusions

CAR-T cells have yet to demonstrate impressive efficacy in thoracic malignancies and safety concerns remain. Heterogeneity in conditioning regimens, disease stage, molecular characteristics of individual tumors, and lines of prior therapy complicates direct efficacy comparisons. As only phase 1 trials have reported results to date, little can be said about the efficacy of CAR-T cells for thoracic malignancies at this time. TCR-T cells have the advantage of also targeting intracellular antigens, but safety concerns and the same issues encountered by CAR-T cells have limited their success to date [17]. TILs are perhaps the simplest yet most eloquent of the cell therapies discussed in this review and are the only therapy that has demonstrated consistent clinical responses in a solid tumor. Challenges in identifying TILs likely to induce antitumor responses have limited their clinical applications in thoracic cancers so far, but novel screening methods may facilitate this process in the near future [5]. The requirement for surgical resection of a portion of the tumor is another major limitation to this therapy modality. NK cells perhaps offer the most versatile platform for an effective cell therapy, as they can be engineered to express a CAR or TCR like T cells, but also have the ability to be used in combination with targeting molecules such as BiKES and TriKES to enable easier targeting of multiple antigens and also have the unique ability to recognize and kill tumor cells which have down-regulated expression of MHCI [18]. This functional redundancy in their ability to recognize tumor cells in a number of ways offers a theoretical benefit over T cell-based therapies and may be less likely to succumb to antigen escape or acquired resistance seen in all cell therapies. Despite these theoretical advantages, no NK cell product has advanced to a phase 3 trial, and much work remains both to identify patients who are likely to benefit and to overcome resistance mechanisms that have limited the success of NK cells in clinical trials to date.

Cell therapies such as CAR-T cells, NK cells, TILs, and TCR-T cells are a promising treatment option that may one day be an option for patients with thoracic malignancies and have the potential to change the outlook of these diseases dramatically. However, this field is still rather new and much more basic science research will be necessary to learn how to design a safe and effective cell therapy product that has a chance to advance to late-stage clinical trials. It should be noted that many of the novel strategies employed in the preclinical studies mentioned in this review are in early stages of development and are not close to being tested in human clinical trials. It is also worth mentioning that cell therapies are currently very costly and only offered at specialized centers. Strategies aimed at reducing costs and streamlining treatment protocols so that these therapies can also be accessed in under-resourced areas will greatly expand the pool of patients that can benefit.

Multigene editing approaches, checkpoint inhibition, conditioning of the TME with radiation or microwave ablation, optimizing timing and dosing of cells, optimizing cytokine support, and other promising strategies described above should be utilized in combination in future trials. Trials to date have used mostly only one or two of these strategies at a time, but it is clear that this is insufficient. While a more detailed discussion is beyond the scope of this review, combination approaches with other emerging cell therapy platforms such as gamma delta T cell therapies and CAR-macrophages are additional promising areas for future research [275,276]. The versatility of the iPSC platform will likely help facilitate the process of designing more robust cell therapies in a large-scale and repeatable fashion [4]. We have outlined the key obstacles that must be addressed for a cell therapy product to succeed for treatment of thoracic malignancies and hope that the many ongoing trials will help us learn how to address these challenges and eventually engineer a successful cell therapy product with efficacy similar to that seen in hematologic malignancies.

## Figures and Tables

**Table 1:** Target antigens for cell therapies against thoracic malignancies.

Lung Cancer				Malignant Pleural Mesothelioma
Surface Antigens		Intracellular Antigens	Other	Surface Antigens
EGFR HER2 CD70 CEA MSLN ROR1 PSCA MUC1 MUC1-C PD-L1 GPC3 FRα B7-H3 MICA/B KDR/VEGFR2 CEACAM5 IL13Rα2	CLDN6 CLDN18.2 c-MET FMR1NB Lewis-Y AXL CD44v6 CD47 CD56 (SCLC) L1CAM/CD171 (SCLC) LunX DLL3 (SCLC) Hsp70 EphA2 PTK7 GD2 (SCLC)	Mutated TP53 CLDN6 CLDN18.2 NY-ESO-1 KRAS mutations MAGE-A antigens KK-LC-1 Patient-specific neoantigens	TGFβ (extracellular) Loss of heterozygosity of HLA alleles	EGFR (ErbB1) HER2 (ErbB2) ErbB3 ErbB4 CD70 MSLN MUC1 FAP IL13Rα2 c-MET

Antigens with preclinical activity against thoracic cancers, explored previously in clinical trials, or under investigation in ongoing clinical trials registered at www.clinicaltrials.gov. Key: EGFR/ErbB1: epidermal growth factor receptor; HER2/ErbB2: human epidermal growth factor receptor 2; CEA: carcinoembryonic antigen; MSLN: mesothelin; ROR1: receptor tyrosine kinase-like orphan receptor 1; MUC1: mucin 1; MUC1-C: mucin 1 cell surface-associated C-terminal antigen; PD-L1: programmed death-ligand 1; PSCA: prostate stem cell antigen; GPC3: glypican-3; FRα: folate receptor-α; GD2: disialoganglioside 2; L1CAM/CD171: neuronal L1 cell adhesion molecule/CD171; FAP: fibroblast activating protein; IL13Rα2: interleukin-13 receptor a2; KDR/VEGFR2: kinase insert domain receptor of vascular endothelial growth factor 2 receptor; CEACAM5: carcinoembryonic antigen-related cell adhesion molecule 5; CD44v6: variant domain 6 of CD44; LunX: lung-specific X; EphA2: erythropoietin-producing hepatocellular carcinoma 2; FMR1NB: fragile X mental retardation 1 neighbor; PTK7: protein tyrosine kinase 7; NY-ESO-1: New York esophageal squamous cell carcinoma 1; MICA/B: Major histocompatibility complex class 1 polypeptide-related sequence A/B; DLL3: delta-like ligand 3; MAGE-A antigens: melanoma-associated antigens; TGFβ: transforming growth factor β; CLDN18.2: Claudin 18.2; CLDN6: Claudin 6; KRAS: Kirsten rat sarcoma; SCLC: small cell lung cancer; NSCLC: non-small cell lung cancer; HLA: human leukocyte antigen; KK-LC-1: Kita-Kyushu lung cancer antigen-1.

**Table 2:** Selected published clinical trials of CAR-T cells, NK cells, TCR-T cells, and TILs as treatments for lung cancer or malignant pleural mesothelioma.

Cell Therapy Product	Cell Source	Target	Disease	Phase	Cell Dose	Conditioning Regimen	Number of Patients	Relevant Findings	Reference, Year
TILs without IL-2	Autologous	N/A	Metastatic lung adenocarcinoma	NR	0.5–4.8 × 10^9^ cells total.	None	N = 7	PR: 0% SD: 71% Trafficking of cells to tumor.	Kradin, 1987 [104]
TILs + continuous infusion of IL-2	Autologous	N/A	NSCLC, melanoma, or renal cell carcinoma	NR	1.5 × 10^9^ cells	None	N = 31	NSCLC PR: 37%	Kradin, 1989 [105]
TILs + IL-2 following surgical resection	Autologous	N/A	Patients with stage 3 NSCLC	Pilot study	4–70 × 10^9^ cells	None	N = 24	mOS—14 months. 2 year OS = 40%	Ratto, 1995 [106]
TIls + IL-2 following surgical resection	Autologous	N/A	Patients with stage 3b NSCLC	Pilot study	4–70 × 10^9^ cells	None	N = 11	mOS = 7.5 months (95% CI, 6.0–9.47 months)	Melioli, 1996 [107]
TILs + recombinant IL-2 following surgery	Autologous	N/A	Phase 2 randomized stage 2, 3a, or 3b NSCLC deemed to have potentially resectable tumors	NR	4–70 × 10^9^ cells	None	N = 113	Total: mOS 22.4 months vs. 14.1 months for control (*p* < 0.05). stage 2 disease: mOS 22.3 months (TIL) vs. 31 months (no treatment) (*p* = 0.56). stage 3a disease: mOS 22 months (TIL + RT group) vs. 9 (chemotherapy + RT) (*p* = 0.06) stage 3b disease mOS 23.9 months (TIL + RT) vs. 7.3 months (chemotherapy + RT) (*p* < 0.01)	Ratto, 1996 [108]
TILs + IL-2 + chemotherapy + RT	Autologous	N/A	Patients with incompletely resected stage 3 NSCLC	2	Median of 22 × 10^9^ cells (range of 11–42 × 10^9^ cells)	Not applicable. Chemotherapy given after TILs	N = 33 enrolled. N = 13 received TILs + IL-2 + cisplatin + etoposide + RT. TILs. N = 20 controls	mPFS: 227 days (TIL) vs. 265 days (control) *p* > 0.53 mOS: 387 days (TIL) vs. 354 days (control), *p* > 0.7	Ratto, 2000 [109]
TILs + IL-2 + nivolumab	Autologous	N/A	metastatic NSCLC	1	Median of 95 × 10^9^ cells (range of 4.3–175 × 10^9^ cells)	Cyclophosphamide and fludarabine	N = 16.	mOS was not reached (ITT analysis including all 20 patients). PR 23%. CR: 12.5% (durable to 1.5 years)	Creelan, 2021 [110]
TILs + IL-2	Autologous	N/A	metastatic NSCLC	2	1.4–53.2 × 10^9^ cells.	Cyclophosphamide and fludarabine	N = 28	ORR = 21.4% (95% CI, 8.3–41.0). Best overall response: complete metabolic response in 1/28 patients,	Schoenfeld, 2024 [111]
CAR-T cells	Autologous	EGFR	Relapsed or refractory NSCLC with >50% EGFR expression	1	Median dose: 0.97 × 10^7^ cells/kg	None (4/11); cyclophosphamide (2/11); pemetrexed, cisplatin, and cyclophosphamide (2/11); and docetaxel, cisplatin, and cyclophosphamide (3/11).	N = 11	PR: 18%.	Feng, 2016 [117]
CAR-T cells delivered intrapleurally	Autologous	FAP	MPM	1	1 × 10^6^ cells/kg intrapleurally	NR	N = 3	PR: 33%	Hiltbrunner, 2021 [118]
Intrapleural CAR-T cells with an inducible caspase 9 switch +pembrolizumab	Autologous	MSLN	MPM, NSCLC and breast cancer.	1	3 × 10^5^ cells/kg to 6 × 10^7^ cells/kg.	1 dose of cyclophosphamide	N = 27	MPM (N = 23): mOS from 17.7 months (95% CI: 13.2-unable to estimate); 1-year OS: 74% (95% CI: 58–94%) MPM + pembrolizumab (N = 18): mOS 23.9 months (95% CI: 14.7 months-unable to estimate); 1-year OS: 83% (95% CI: 68–100%) PR: 12.5%	Adusumilli, 2021 [119]
CAR-T cells	Autologous	EGFR	Advanced relapsed or refractory NSCLC	1	1 × 10^6^ cells/kg or 3 × 10^6^ cells/kg.	NR	N = 9	mPFS: 7.13 months (95% CI: 2.71–17.1). OS: 15.63 months (95% CI: 8.82–22.03). PR: 11%.	Zhang, 2021 [120]
CAR-T cells (PD-1 knockout)	Autologous	MUC1	Advanced NSCLC	1	Starting dose of 2.5 × 10^6^ cells/kg.	NR	N = 20	PR: 0%	Lin, 2019 [121]
CAR-T cells with truncated EGFR	Autologous	ROR1	ROR1+ NSCLC and TNBC with >20% expression of ROR1 on IHC	1	3.3 × 10^5^ cells/kg to 1 × 10^7^ cells/kg.	Cyclophosphamide	N = 5	PR: 0%	Specht, 2018 [122]
CAR-T cells	Autologous	DLL3	Relapsed or refractory SCLC	1	3 patients received 3 × 10^5^ to 1 × 10^6^ cells/kg.	NR	N = 4	PR: 25% mOS = 7.4 months (range of 4.6–18.9 months) mPFS = 3.7 months (range of 1.1–6.7 months)	Byers, 2022 [39]
TRuC-T cells	Autologous	MSLN	Advanced MSLN-expressing solid tumors, MPM	1/2	5 × 10^7^ cells/m^2^ to 5 × 10^8^ cells/m^2^.	Cyclophosphamide and fludarabine	N = 30 received TRuC-T cells MPM n-23	mPFS = 5.6 months (95% CI, 3.1–5.8 months). mOS = 10.6 months (95% CI, 6.6–15.6 months). ORR = 20%	Hassan, 2023 [126]
NK cells	allogeneic, derived from two relative donors.	N/A	Advanced NSCLC	1	Median dose: 4.15 × 10^6^ cells/kg.	NK infusion 2 days after chemotherapy	N = 15	2/15 had a PR, mPFS was 5.5 months (range of 1–22 months) mOS was 15 months (range of 2–26 mon 1yr OS: 56% 2yr OS: 25%.	Iliopoulou, 2010 [135]
NK cells + docetaxel	Autologous	N/A	Advanced NSCLC	2a	Mean cell count per infusion: 2 × 10^9^ cells.	docetaxel 35 mg/m^2^ was to be given on day 1 and day 8	N = 19.	PR: 10.5%. Median PFS: 2.9 months (range of 1.3–6.4 months).	Yang, 2013 [136]
NK cells	NK-92 cell line	N/A	Advanced cancers, including lung cancer	1	1 × 10^9^ cells/m^2^ to 1 × 10^10^ cells/m^2^.	None	N = 15	PR: 0%	Tonn, 2013 [137]
NK cells + cryoablation vs. cryoablation only	Allogeneic	N/A	Advanced NSCLC	NR	2 total NK cell infusions (cells/dose NR) were given on days 13–15 following CT-guided percutaneous cryoablation of tumors on day 9 and day 12.	NR	N = 60	PR: 63.3% in the NK cells + cryoablation group vs. 43.3% in the cryoablation only (*p* < 0.01). CR: 23% NK cells + percutaneous cryoablation group vs. 13% cryoablation only (*p* > 0.05).	Lin, 2017 [138]
NK cells stimulated ex vivo with low-dose IL-2 and mHsp70	Autologous	Hsp70	Unresectable stage IIIa/b NSLC with mHsp70-positive tumors after chemoRT vs. chemoRT alone	2	1.04 × 10^8^ cells to 5.63 × 10^8^ cells.	NR	N = 13	1 year probability of PFS: 67% (95% CI, 19–90%) in the NK cell group vs. 33% (95% CI, 5–68%), *p*-value = 0.36. iCR: 16% vs. 16% (control) iPR: 16% vs. 16% (control)	Multhoff, 2020 [60]
NK cells + sintilimab	Autologous	N/A	Advanced NSCLC	Pilot study	3 × 10^9^ cells every 3 weeks. Sintilimab was given with NK cells every 3 weeks.	NR	N = 20	Median PFS: 11.6 months (range of 1.33–25.9+ months). Median OS: 17.7 months (range of 2.3–25.9+ months). PR: 40% CR: 5%	Jia, 2022 [139]
NK cells + pembrolizumab vs. Pembrolizumab alone	Allogeneic	N/A	Advanced NSCLC patients PDL1 > 1%	NR	NR.	NR	N = 109.	NK m OS: 15.5 mo vs. pembro mOS: 13.3 months. NK PFS: 6.5 months vs. pembro PFS: 4.3 months. ORR 36.4% vs. 18.5% *p* < 0.05	Lin [140] 2020
NK cells + pembrolizumab vs. pembrolizumab	Autologous	N/A	Advanced NSCLC with PDL1 > 1%	1 and pilot phase 2a	2 × 10^9^ cells/dose or 4 × 10^9^ cells/dose.	NR	N = 18	ORR 41% NK/Pembro vs. 0% pembro (*p*-value = 0.11). mPFS NK/Pembro 6.2 months (95% CI, 1.4 months-not listed) vs. Pembro 1.6 months (95% CI, 0.6–4.7 months) (*p*-value = 0.001). Survival rate at 1 year OS—66.7% NK/pembro compared to 50% pembro (*p*-value = 0.39).	Kim, 2022 [141]
NK cells + IL-2 + ICI	allogeneic iPSC-derived, “off the shelf”	N/A	NSCLC and classical Hodgkin’s lymphoma	1	3 × 10^8^ NK cells and 6 MIU subcutaneous IL-2.	Cyclophosphamide and fludarabine	N = 12	PR: 16%	Patel, 2022 [143]
TCR-T cells	Autologous	Driver mutations in EGFR,KRAS, or TP53	Multiple solid tumors with driver mutations in KRAS, TP53, or EGFR.	1/2	0.1–1 × 10^10^ cells, 1–7 × 10^10^ cells, and 7–15 × 10^10^ cells.	NR	N = 3	Patient 1 (NSCLC): PR, PFS of 6 months	Morelli, 2023 [131]
TCR-T cells	Autologous	MAGE-A4	Relapsed or refractory solid tumors that express MAGE-A4 and have the HLA-A*02 allele	1	0.08–0.12 × 10^9^ cells, 0.5–1.2 × 10^9^ cells, and 1.2–10 × 10^9^ cells.	Cyclophosphamide and fludarabine	N = 38.	ORR: 24% (95% CI, 11.4–40.2%); mPFS: 12.4 weeks (95% CI, 10.9–19.1 weeks); mOS: 42.9 weeks (95% CI, 20.7 weeks-not achieved).	Hong, 2023 [130]
TCR-T cells	Autologous	MAGE-A10	advanced NSCLC expressing MAGE-A10 and HLA-A*02:01 allele ± the HLA-A*02:06 allele	1	0.08–0.12 × 10^9^ cells, 0.5–1.2 × 10^9^ cells, and 1.2–15 × 10^9^ cells.	Cyclophosphamide and fludarabine	N = 7	PR: 14% mPFS: 58 days (range of 1 to 89 days) mOS: 132 days (range of 10 to 458 days)	Blumenschein, 2022 [129]

Key: AE: adverse event; EGFR: epidermal growth factor receptor; MUC-1: mucin 1; NSCLC: non-small cell lung cancer; MPM: malignant pleural mesothelioma; T cell receptor fusion construct (TRuC); PFS: progression-free survival; CI: confidence interval; OS: overall survival; SD: stable disease; PR: partial response; PD: progressive disease; FAP: fibroblast activating protein; ORR: objective response rate; PD-1: programmed cell death-1; PD-L1: programmed cell death-ligand 1; TNBC: triple negative breast cancer; IHC: immunohistochemistry; NK cells: natural killer cells; iPSC: induced pluripotent stem cells; IL-2: interleukin-2; IL-15: interleukin-15; ICI: immune checkpoint inhibitor; MIU: million international units; iPR: immune partial response; iSD: immune stable disease; GVHD: graft-versus-host disease; ITT: intention to treat; ORR: objective response rate; DCR: disease control rate; MSLN: mesothelin; mHsp70: membrane-bound Hsp70; MAGE: melanoma-associated antigen; SEM: standard error of the mean; N/A: no specific target or not specified; RT: radiotherapy; NR: not specified or not reported.

**Table 3:** Actively recruiting clinical trials testing cell therapies for lung cancer or mesothelioma available on clinicaltrials.gov.

NCT Identifier	Cell Therapy Product	Cell Source	Planned Dose	Disease(s)	Target	Phase	Sponsor
NCT05341492	CAR-T cells	NR	2 × 10^6^ cell/kg	EGFR/B7H3+ solid tumors, including lung cancer	EGFR/B7H3	1	Second Affiliated Hospital of Guangzhou Medical University
NCT03198052	CAR-T cells	Autologous	1 × 10^6^ to 1 × 10^10^ cells/kg	Patients with advanced lung cancer or other cancers expressing an antigen of interest	CD8+ T cell targets will include MSLN, GPC3, Claudin 18.2, GUCY2C, B7-H3, PSCA, MUC1, PSMA, TGFβ, HER2, Lewis-Y, AXL, and EGFR. TGF-β CAR inserted	1	The First Affiliated Hospital of Sun Yat-sen University, Guangzhou, Guangdong, China
NCT05060796	CAR-T cells	Autologous	0.5 × 10^6^ cells/kg, 1.58 × 10^6^ cells/kg, and 5 × 10^6^ cells/kg	Advanced NSCLC	CXCR-modified CAR-T cells targeting EGFR	1	Second Affiliated Hospital of Guangzhou Medical University
NCT05620342	CAR-T cells	Autologous	NR	ES-SCLC or Stage IV NSCLC	GD2 IL-15 expression inducible caspase9 safety switch.	1	University of North Carolina Lineberger Comprehensive Cancer Center
NCT05680922	CAR-T cells	Autologous	NR	Extensive stage SCLC or lung large cell neuroendocrine carcinoma	DLL3	1	Legend Biotech USA Inc.
NCT06051695	CAR-T cells	Autologous	NR	MSLN expressing solid tumors with LOH of HLA-A*02	MSLN with LOH of HLA-A*02	1/2	A2 Biotherapeutics Inc.
NCT05736731	CAR-T cells	Autologous	NR	Solid tumors that express CEA and LOH of HLA-A*02	CEA and LOH of HLA-A*02	1/2	A2 Biotherapeutics Inc.
NCT05274451	CAR-T cells	Autologous	4 dose levels (NR)	ROR1+ relapsed or refractory NSCLC or TNBC	ROR1	1	Lyell Immunopharma
NCT03198546	CAR-T cells	Autologous	NR	HCC or sqNSCLC with GPC3 expression	GPC3 and TGF-β	1	Second Affiliated Hospital of Guangzhou Medical University
NCT05035407	TCR-T cells + aldesleukin (IL-2), cyclophosphamide + fludarabine	Autologous	1 × 10^8^ cells	NSCLC and solid tumors that are KK-LC-1 positive. HLA-A01:01	KK-LC-1	1	National Cancer Institute
NCT05483491	TCR-T cells + aldesleukin, cyclophosphamide + fludarabine	Autologous	NR	NSCLC and KK-LC-1-positive solid tumors. HLA A*01:01.	KK-LC-1	1	Christian Hinrichs
NCT05239143	CAR-T cells with inducible safety switch and conditioning chemotherapy	Allogeneic	NR	Multiple solid tumors with MUC1-C expression, including NSCLC	MUC1-C	1	Poseida Therapeutics, Inc.
NCT04952272	Tumors will be injected with type A/C CpG-ODN ± CAR-T cells which secrete anti-OX40 scFv	NR	NR	Advanced solid tumors	CAR-T cells will be engineered to secrete anti-OX40 scFv	1	Second Affiliated Hospital of Guangzhou Medical University
NCT03740256	CAR-T cells + intratumoral injection of an oncolytic adenovirus	Autologous	Multiple dose levels to be used, from 0 to 1 × 10^8^ cells	Advanced/refractory HER2-positive solid tumors, including lung cancer	HER2	1	Baylor College of Medicine
NCT06043466	CAR-T cells preceded by conditioning with cyclophosphamide + fludarabine	Autologous	2–10 × 10^6^ cells/kg	Multiple CEA-positive advanced solid tumors, including NSCLC	CEA	1	Chongqing Precision Biotech Co., Ltd.
NCT06010862	CAR-T cells preceded by conditioning with cyclophosphamide + fludarabine	Autologous	1–10 × 10^6^ cells/kg IV or IP	Multiple CEA-positive advanced solid tumors, including NSCLC	CEA	1	Chongqing Precision Biotech Co., Ltd.
NCT06126406	CAR-T cells preceded by conditioning with cyclophosphamide + fludarabine	Autologous	IV at 3–10 × 10^6^ cells/kg or IP at 1–10 × 10^6^ cells/kg	Multiple CEA-positive advanced solid tumors, including lung cancer	CEA	1	Chongqing Precision Biotech Co., Ltd.
NCT06006390	CAR-T cells preceded by conditioning with cyclophosphamide + fludarabine	Autologous	1–10 × 10^6^ cells/kg IV or IP	Multiple CEA-positive advanced solid tumors, including lung cancer	CEA	1/2	Chongqing Precision Biotech Co., Ltd.
NCT04577326	CAR-T cells preceded by conditioning with cyclophosphamide.	Autologous	Intrapleural administration up to 3 × 10^7^ cells/kg	Malignant pleural mesothelioma	MSLN	1	Memorial Sloan Kettering Cancer Center
NCT04503278	CAR-T cells will be given either with or without a CLDN6 RNA vaccine contained within lipoplexes	NR	NR	Advanced CLDN-6-positive solid tumors, including NSCLC	CLDN6	1	BioNTech Cell and Gene Therapies GmbH
NCT06256055	CAR-T cells produced via a circular mRNA which encodes an anti-mesothelin CAR	NR	4 weekly injections of 1 × 10^8^ cells to 2 × 10^9^ cells.	Advanced MSLN-positive solid tumors, including mesothelioma.	MSLN	1	UTC Therapeutics Inc.
NCT05795595	CAR-T cells produced ex vivo via CRISPR Cas9	Allogeneic	NR	Multiple advanced solid tumors, including malignant pleural mesothelioma	CD70	1/2	CRISPR therapeutics AG
NCT05120271	CAR-T cells preceded by conditioning with cyclophosphamide + fludarabine	Autologous	5 dose levels (doses NR)	Advanced GPC3-positive solid tumors, including squamous cell carcinoma of the lung.	GPC3	1/2	Sotio Biotech Inc.
NCT05568680	CAR-T cells preceded by non-myeloablative lymphodepleting chemotherapy	Autologous	NR	Advanced mesothelin-positive tumors, including mesothelioma	MSLN	1	Verismo Therapeutics
NCT06241456	CAR-T cells will be given following chemotherapy with cyclophosphamide, fludarabine OR cisplatin, and docetaxel. CAR-T cells will be given with or without cetuximab.	Allogeneic	NR	HER2-positive advanced solid tumors	HER2	1	Fate Therapeutics
NCT04119024	CAR-T cells preceded by lymphodepletion with cyclophosphamide + fludarabine	Autologous	NR	NSCLC and several other advanced solid tumors	IL13Rα2	1	Jonsson Comprehensive Cancer Center
NCT05366478	TILs + IL-2	Autologous	Single dose (dose NR)	Advanced solid tumors, including NSCLC	N/A	1	Suzhou BlueHorse Therapeutics Co., Ltd.
NCT04614103	TILs + IL-2	Autologous	NR	Metastatic stage IV NSCLC	N/A	2	Iovance Biotherapeutics, Inc.
NCT05878028	TILs + Tislelizumab + docetaxel	Autologous	3–10 × 10^9^ cells/m^2^ for 6 cycles	Advanced NSCLC resistant to PD-1 ICI	N/A	2	Quanli Gao
NCT05681780	TILs with CD40L stimulation + nivolumab + IL-2 + lymphodepletion with cyclophosphamide + fludarabine	Autologous	NR	Stage IV or recurrent NSCLC with ALK, ROS1, EGFR, or ERBB2 genetic alterations.	N/A	1/2	H. Lee Moffitt Cancer Center and Research Institute
NCT03645928	TILs preceded by nonmyeloablative lymphodepletion and followed by IL-2 administration alone or in combination with nivolumab, ipilimumab, or pembrolizumab	Autologous	NR	Advanced NSCLC, melanoma, or head and neck squamous cell carcinoma	N/A	2	Iovance Biotherapeutics, Inc.
NCT05573035	TILs which have been epigenetically reprogrammed	Autologous	NR	Advanced NSCLC, melanoma, or colorectal cancer	N/A	1	Lyell Immunopharma, Inc.
NCT05902520	TILs which co-express CD39 and 103 preceded by lymphodepleting chemotherapy and followed by IL-2. TILs in one arm of the study will be expanded in vitro with a siRNA meant to decrease PD-1 expression	Autologous	1–40 × 10^9^ cells for a single infusion	Advanced solid tumors, including lung cancer	N/A	1	AgonOx, Inc.
NCT05576077	TILs + pembrolizumab, IL-2, and low-dose radiation therapy. Patients will also receive nonmyeloablative lymphodepleting chemotherapy prior to TIL infusion.	Autologous	NR	Advanced solid tumors, including NSCLC	N/A	1	Turnstone Biologics, Corp.
NCT06060613	TILs which express membrane-bound IL-15 preceded by lymphodepletion with cyclophosphamide + fludarabine and followed by acetazolamide	Autologous	NR	Advanced solid tumors, including lung cancer	N/A	1/2	Obsidian Therapeutics, Inc.
NCT06237881	TILs which have been engineered with SOCS1 gene inactivation and will be preceded with lymphodepletion with cyclophosphamide + fludarabine and followed by IL-2	Autologous	NR	Advanced solid tumors, including lung cancer	N/A	1/2	M.D. Anderson Cancer Center
NCT05361174	TILs engineered with PDCD1 inactivation preceded by lymphodepletion with cyclophosphamide + fludarabine	Autologous	NR	Stage 3 or 4 NSCLC or unresectable or metastatic melanoma	N/A	1/2	Iovance Biotherapeutics, Inc.
NCT06235242	TILs + teraplizumab	Autologous	NR	NSCLC	N/A	Single arm, open design. Phase NR	Grit Biotechnology
NCT02133196	TILs + IL-2 preceded by nonmyeloablative lymphodepletion with cyclophosphamide + fludarabine	Autologous	NR	Advanced NSCLC	N/A	2	National Cancer Institute
NCT03935893	TILs + IL-2 preceded by nonmyeloablative lymphodepletion with cyclophosphamide + fludarabine	Autologous	1 × 10^9^ cells to 2 × 10^11^ cells	Multiple advanced solid tumors, including mesothelioma	N/A	2	Udai Kammula (University of Pittsburgh)
NCT06375187	TILs preceded by lymphodepletion (NR) and proceeded by anti-PD1 antibody	Autologous	NR	Advanced lung cancer and other advanced solid tumors	N/A	1	Shanghai Juncell Therapeutics
NCT06491225	TILs	Autologous	NR	Advanced lung adenocarcinoma	N/A	1	Grit Biotechnology
NCT03778814	TCR-T cells	Autologous	To be given intravenously, intraaterially, or intratumorally. Dose NR	Advanced lung cancer and other solid tumors	KK-LC-1 or other	1	Second Affiliated Hospital of Guangzhou Medical University
NCT06043713	TCR-T cells preceded by lymphodepletion with cyclophosphamide + fludarabine or bendamustine	Autologous	NR	Metastatic NSCLC, pancreatic or colorectal cancer with a KRAS G12V mutation, HLA-A*11:01 allele-restricted.	KRAS G12V	1	Fred Hutchinson Cancer Center
NCT06218914	TCR-T cells preceded by nonmyeloablative lymphodepletion with cyclophosphamide + fludarabine and followed by recombinant IL-2	Autologous	NR	Multiple advanced solid tumors with KRAS G12D mutation, including NSCLC. HLA-C*0:02 allele-restricted.	KRAS G12D	1	Neogene Therapeutics, Inc.
NCT05877599	TCR-T cells preceded by nonmyeloablative lymphodepletion with cyclophosphamide + fludarabine and followed by recombinant IL-2	Autologous	NR	Multiple advanced solid tumors with TP53 R175H mutation, including NSCLC. HLA-A*02:01 allele-restricted	TP53 R175H	1	Neogene Therapeutics, Inc.
NCT03412877	TCR-T cells + pembrolizumab preceded by lymphodepletion with cyclophosphamide + fludarabine and followed by aldesleukin (IL-2)	Autologous	NR	Multiple metastatic solid tumors, including NSCLC.	Neoantigen specific to each patient	2	National Cancer Institute
NCT06253520	TCR-T cells + a vaccine against the same KRAS mutation-related antigens preceded by lymphodepletion with cyclophosphamide + fludarabine and followed by aldesleukin (IL-2)	Autologous	NR	Multiple metastatic solid tumors with either KRAS G12D mutation or KRAS G12V mutation, including NSCLC.	KRAS mutations	1	National Cancer Institute
NCT05296564	TCR-T cells preceded by lymphodepletion with cyclophosphamide + fludarabine and followed by aldesleukin (IL-2)	NR	1 × 10^9^ cells, 5 × 10^9^ cells, and 1 × 10^10^ cells	Multiple metastatic solid tumors with expression of NY-ESO-1, including NSCLC.	NY-ESO-1	1/2	Hadassah Medical Organization
NCT06105021	TCR-T cells preceded by lymphodepleting chemotherapy	Autologous	NR	Advanced or metastatic cancers with a KRAS G12V mutation. HLA-A*11:01 allele-restricted	KRAS G12V	1/2	Affini-T Therapeutics, Inc.
NCT03420963	NK cells to be given with etoposide + cyclophosphamide	Allogeneic	NR	Children or young adults with advanced solid tumors	N/A	1	M.D. Anderson Cancer Center

Selected actively recruiting interventional clinical trials on www.clinicaltrials.gov investigating the use of CAR-T cells, TILs, TCR-T cells, or NK cells in lung cancer or malignant pleural mesothelioma (last searched on 10 July 2024). Information on the administration of lymphodepleting chemotherapy and cytokine support is provided when this information was available. Key: CAR-T cells: chimeric antigen receptor T cells; TILs: tumor-infiltrating lymphocytes; NK cells: natural killer cells; TCR-T cells: T cell receptor-engineered T cells; PD-1: programmed cell death-1; PD-L1: programmed cell death-ligand 1; NSCLC: non-small cell lung cancer; SCLC: small cell lung cancer; TNBC: triple negative breast cancer; HCC: hepatocellular carcinoma; MUC1-C: mucin 1 cell surface-associated C-terminal antigen; GPC3: glypican-3; MSLN: mesothelin; CLDN6: Claudin 6; CLDN18.2: Claudin 18.2; IL-2: interleukin-2; *CISH*: the gene coding for cytokine-inducible SH2-containing protein (CIS); KRAS: Kirsten rat sarcoma; IL13Rα2: interleukin-13 receptor a2; IV: intravenous; IP: intraperitoneal; NR: not specified or not reported; N/A: no specific target.
